# Transcriptome fine-mapping in *Fusobacterium nucleatum* reveals FoxJ, a new σ^E^-dependent small RNA with unusual mRNA activation activity

**DOI:** 10.1128/mbio.03536-23

**Published:** 2024-03-04

**Authors:** Falk Ponath, Yan Zhu, Jörg Vogel

**Affiliations:** 1 Helmholtz Institute for RNA-based Infection Research (HIRI), Helmholtz Centre for Infection Research (HZI), Würzburg, Germany; 2 RNA Biology Group, Institute for Molecular Infection Biology (IMIB), University of Würzburg, Würzburg, Germany; Institut Pasteur, Paris, France

**Keywords:** RNA-seq, noncoding RNA, small RNA, post-transcriptional control, extracytoplasmic sigma factor, envelope stress

## Abstract

The oral commensal *Fusobacterium nucleatum* can spread to extra-oral sites, where it is associated with diverse pathologies, including pre-term birth and cancer. Due to the evolutionary distance of *F. nucleatum* to other model bacteria, we lack a deeper understanding of the RNA regulatory networks that allow this bacterium to adapt to its various niches. As a first step in that direction, we recently showed that *F. nucleatum* harbors a global stress response governed by the extracytoplasmic function sigma factor, σ^E^, which displays a striking functional conservation with Proteobacteria and includes a noncoding arm in the form of a regulatory small RNA (sRNA), FoxI. To search for putative additional σ^E^-dependent sRNAs, we comprehensively mapped the 5′ and 3′ ends of transcripts in the model strain ATCC 23726. This enabled the discovery of FoxJ, a ~156-nucleotide sRNA previously misannotated as the 5′ untranslated region (UTR) of *ylmH*. FoxJ is tightly controlled by σ^E^ and activated by the same stress conditions as is FoxI. Both sRNAs act as mRNA repressors of the abundant porin FomA, but FoxJ also regulates genes that are distinct from the target suite of FoxI. Moreover, FoxJ differs from other σ^E^-dependent sRNAs in that it also positively regulates genes at the post-transcriptional level. We provide preliminary evidence for a new mode of sRNA-mediated mRNA activation, which involves the targeting of intra-operonic terminators. Overall, our study provides an important resource through the comprehensive annotation of 5′ and 3′ UTRs in *F. nucleatum* and expands our understanding of the σ^E^ response in this evolutionarily distant bacterium.

**IMPORTANCE:**

The oral microbe *Fusobacterium nucleatum* can colonize secondary sites, including cancer tissue, and likely deploys complex regulatory systems to adapt to these new environments. These systems are largely unknown, partly due to the phylogenetic distance of *F. nucleatum* to other model organisms. Previously, we identified a global stress response mediated by σ^E^ that displays functional conservation with the envelope stress response in Proteobacteria, comprising a coding and noncoding regulatory arm. Through global identification of transcriptional start and stop sites, we uncovered the small RNA (sRNA) FoxJ as a novel component of the noncoding arm of the σ^E^ response in *F. nucleatum*. Together with its companion sRNA FoxI, FoxJ post-transcriptionally modulates the synthesis of envelope proteins, revealing a conserved function for σ^E^-dependent sRNAs between Fusobacteriota and Proteobacteria. Moreover, FoxJ activates the gene expression for several targets, which is a mode of regulation previously unseen in the noncoding arm of the σ^E^ response.

## INTRODUCTION

The anaerobic microbe *Fusobacterium nucleatum* is a major constituent of the oral microbiome, and it is linked to dental plaque formation (1, 2). *F. nucleatum* is also implicated in different pathologies such as periodontitis, adverse pregnancy outcomes, or different cancers (3–7). Despite its growing medical importance, a deeper understanding of gene regulation mechanisms in this bacterium is lacking due to its early evolutionary divergence from the current bacterial model species. Prior to the recent development of a broad set of genetic tools (8, 9) and improvements in plasmid delivery (10), the limited genetic accessibility of *F. nucleatum* was an additional constraint for functional studies.

Considering the different oral and extraoral niches colonized by *F. nucleatum*, it is evident that the bacterium senses and adapts to changing environmental conditions. Recent work showed that *F. nucleatum* relays environmental cues through the two-component systems CarRS and ModRS involved in interspecies coaggregation and resistance to hydrogen peroxide, respectively (11, 12). Additionally, *F. nucleatum* harbors a global stress response coordinated through the extracytoplasmic function sigma factor (ECF) sigma E (σ^E^), encoded by the *rpoE* gene (8).

The σ^E^-mediated stress response is well understood in Proteobacteria such as *Escherichia coli, Salmonella enterica,* and *Vibrio cholerae,* where it relies on two regulatory principles. First, the transcriptional activation of genes involved in maintaining membrane homeostasis via σ^E^ directly (the “coding arm” of the response). Second, the upregulation of σ^E^-dependent small RNAs (sRNAs) that act to post-transcriptionally repress outer membrane protein (OMP) synthesis (the “noncoding arm” of the response) (13–15). In *E. coli* and *Salmonella*, the noncoding arm consists of up to three sRNAs MicA, RybB, and MicL (16–22). MicA and RybB regulate the expression of all major outer membrane porins, with several shared targets such as OmpA and others that are unique to each sRNA (14). MicL primarily targets the abundant lipoprotein Lpp (17), although recent global interactome studies suggest additional targets for this sRNA (23, 24).

Based on the recent identification of a large suite of conserved fusobacterial sRNAs (25), sRNA-mediated post-transcriptional regulation is likely to play an important role in *F. nucleatum*. Part of this suite of sRNAs is FoxI, which inhibits the translation of several abundant envelope-associated proteins such as FomA and MglB (8, 25). Transcriptionally activated by σ^E^, FoxI seems to function as the noncoding arm of this stress response in *F. nucleatum* (8). This highlights a striking conservation of the regulatory principles of the σ^E^-response across evolutionary distant phyla. In light of the early phylogenetic divergence of Fusobacteriota from all other bacteria (26), it also suggests that this regulatory principle arose early during bacterial evolution.

It was puzzling that there was only a single σ^E^-dependent sRNA in *F. nucleatum*, in contrast to the division of labor by two or three sRNAs in Proteobacteria, as discussed above. Interestingly, the deletion of FoxI did not fully abrogate the negative regulation observed upon σ^E^ activation (8), which we considered another hint at the existence of additional σ^E^-dependent sRNAs. Yet, inspection of the promoter regions of the ~24 annotated *F. nucleatum* sRNAs showed no obvious σ^E^-binding site except for the promoter of FoxI.

Thus, to discover additional sRNAs that might have been overlooked due to limited genome annotation, we expanded our recent mapping of the primary transcriptome of *F. nucleatum* subsp. *nucleatum* ATCC 25586 to the widely used and genetically tractable strain ATCC 23726. In addition to the 5′ ends of transcripts, we also mapped their 3′ ends. Taking advantage of this comprehensive annotation, we have uncovered a second σ^E^-dependent sRNA in *F. nucleatum*, which has an overlapping but also distinct targetome compared to FoxI; we will refer to this sRNA as FoxJ. Moreover, we show that FoxJ is unique among the known σ^E^-dependent sRNAs, because it activates multiple mRNAs, in addition to the common function as mRNA repressor. Positive regulation seems to rely on one or several novel mechanisms that involve the targeting of internal terminator regions in operons. Our results expand our understanding of the noncoding arm of the fusobacterial σ^E^-response and reinforce the functional conservation of this regulatory principle between evolutionary distant phyla.

## RESULTS

### High-resolution RNA-based genome annotation of *F. nucleatum*

We previously generated high-resolution global RNA maps for five clinically relevant fusobacterial strains, including the reference strain *F. nucleatum* subsp. *nucleatum* ATCC 25586 (25). However, like most fusobacterial isolates, this strain was genetically intractable until recently (10). Therefore, gene function studies have focused mostly on the genetically tractable strain ATCC 23726, which is closely related to the reference strain (98.7% nucleotide identity [27]). Moreover, the recent development of genetic tools and improvements in transformation have also focused on this strain (9, 10, 28). We therefore decided to fine-map and annotate the transcriptome of strain ATCC 23726.

As a first step, we performed differential RNA-seq (dRNA-seq) to globally identify transcriptional start sites (TSSs) and 5′ untranslated regions (UTRs) following our established protocol (25, 29). Based on the enzymatic removal of processed RNA carrying a 5′ monophosphate by terminator 5′-phosphate-dependent Exonuclease (Tex) (29), dRNA-seq enriches unprocessed RNA species that are marked by a 5′ triphosphate. This enrichment of primary transcripts enables the identification of TSSs and associated 5′ UTRs.

**Fig 1:**
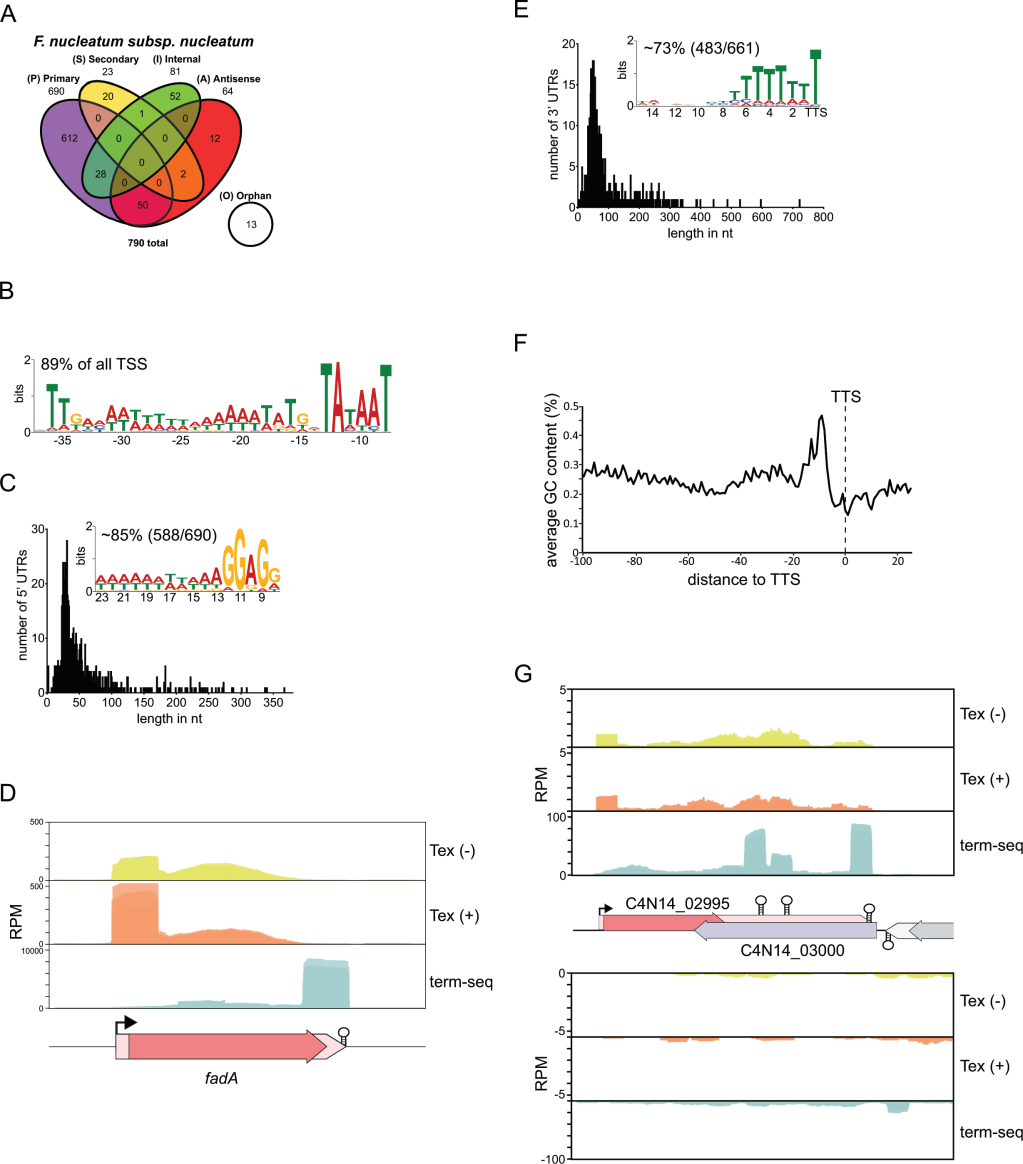
Overview of the global 5′- and 3′-end mapping results for *F. nucleatum*. (**A**) Venn diagram showing the number of detected TSSs for each RNA class. The lower panel shows TSS classification based on expression strength and genomic location. (**B**) Representation of the identified promoter motif found upstream of ~89% of pTSSs. (**C**) Length distribution and corresponding count of all 5′ UTRs associated with pTSSs. The inlet displays the consensus Shine-Dalgarno sequence associated with ~85% of 5′ UTRs and its average distance from the start codon. (**D**) Normalized coverage (by reads per million, RPM) of the dRNA-seq and term-seq libraries for the *fadA* mRNA. The TSS and TTS are indicated by an arrow and stem-loop symbol, respectively. (**E**) Length distribution and corresponding count of all 3′ UTRs associated with TTSs. The inlet displays a consensus sequence found within a 15 nt window upstream of the TTSs. (**F**) Display of the average GC content 100 nt upstream and 24 nt downstream of the TTS. (**G**) Normalized coverage (RPM) of the dRNA-seq and term-seq libraries for the mRNAs of C4N14_02995 and C4N14_03000. The TSS and TTSs are indicated by an arrow and stem-loop, respectively.

Applied to bacteria in the mid-exponential growth phase, dRNA-seq identified a total of 790 TSSs (Fig. 1A), the majority of which (~87%) were primary TSSs (pTSSs), as indicated by their strong enrichment in the Tex-treated libraries. pTSSs mark the main start of a gene or operon, as previously seen with other fusobacterial strains (25). A comparison of the dRNA-seq between the *F. nucleatum* subsp. *nucleatum* strains ATCC 23726 and ATCC 25586 revealed 535 shared homolog genes with detected pTSSs in both data sets, in line with the high similarity between the two strains (Table S1A). Moreover, the upstream regions of ~89% of all pTSSs harbor a common promoter motif with a pronounced −10 box and weaker −35 box (Fig. 1B), which is also found in other fusobacterial strains (25). Similarly, the 5′ UTR length with a median of 39 nt and a prominent Shine-Dalgarno sequence is very similar to other members of this genus (Fig. 1C) (25).

While dRNA-seq maps 5′ UTRs, it does not provide a good annotation of 3′ UTRs (30). In contrast, term-seq enables the determination of native 3′ ends through an initial 3′ end adapter ligation step prior to RNA shearing during library preparation (30). We performed term-seq to globally determine the 3′ UTRs in *F. nucleatum* using the same RNA samples as used for the dRNA-seq analysis. This sequencing approach uncovered 663 transcriptional termination sites (TTSs) across the genome as exemplified for the *fadA* transcript (Fig. 1D; Table S1B). The majority of 3′ UTRs display an increased GC content and a poly-U stretch at their 3′ end (Fig. 1E and F), indicative of the formation of stable stem-loop structures. Such stem-loop structures play an important role in intrinsic transcription termination, an alternative mechanism to Rho-dependent termination (31). The high prevalence of possible stem-loops in the 3′ end of mRNAs suggests a central role of intrinsic transcription termination in *F. nucleatum*.

The 3′ UTRs in *F. nucleatum* display a median length of ~62 nt. Only 16 3′ UTRs were longer than 300 nt, with the 3′ UTR of a glutathione peroxidase (C4N14_02995) gene standing out as the longest (~722 nt). Interestingly, the 3′ UTR of the C4N14_02995 mRNA is transcribed antisense to the downstream gene C4N14_03000, an energy-coupling factor transporter, for which only a few reads could be detected (Fig. 1G). This suggests a possible transcriptional regulation through antisense transcription for this specific gene, which is a common principle of regulation in prokaryotes (32). The regulation of the downstream transporter through antisense transcription is further supported by the conservation of the gene arrangement for C4N14_02995 and C4N14_03000 in different fusobacterial strains (Fig. S1A).

### Sequencing-guided discovery of the sRNA FoxJ

**Fig 2:**
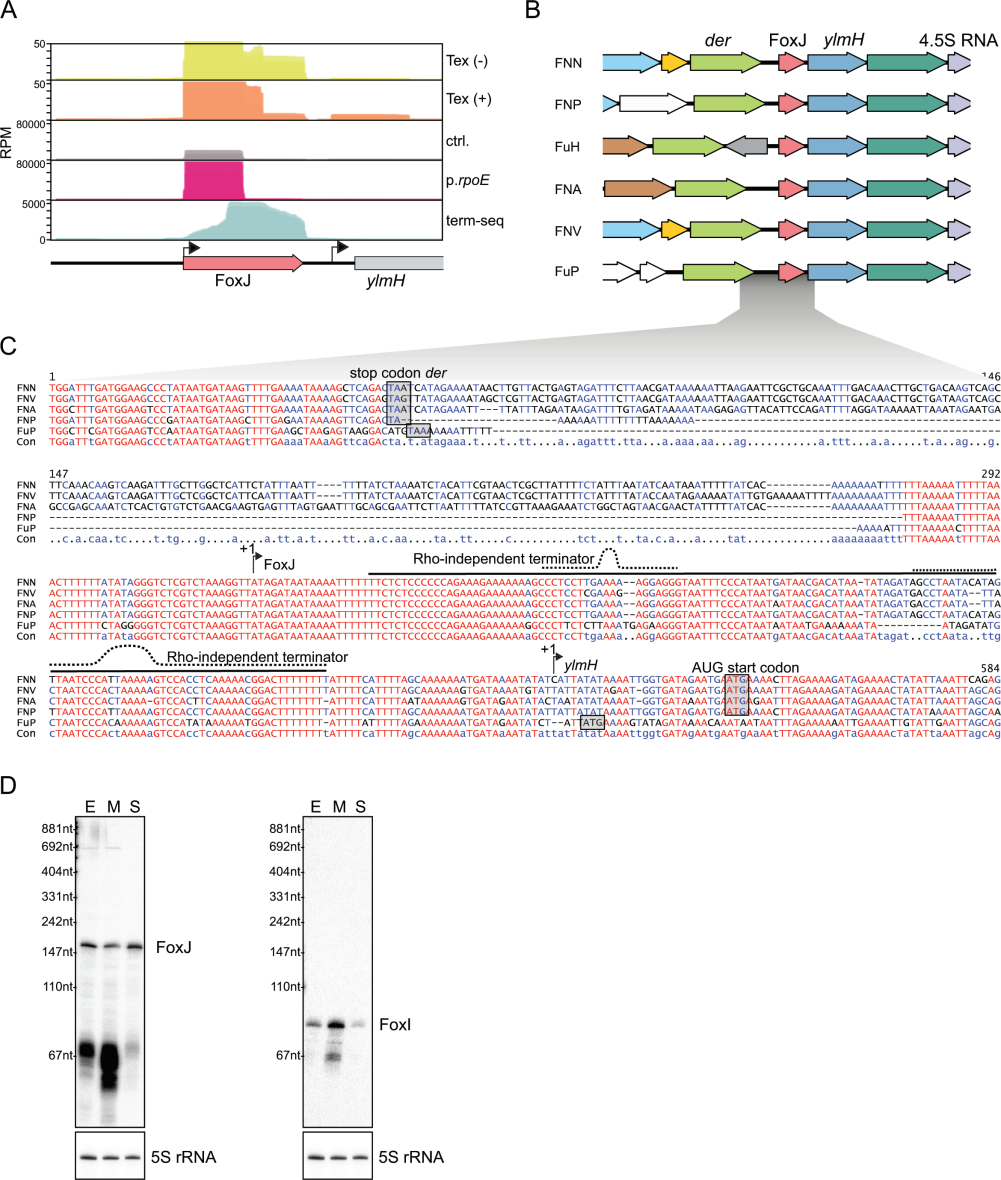
Discovery of the sRNA FoxJ. (**A**) Normalized coverage (reads per million) of the dRNA-seq, term-seq, and libraries from reference (8) (GSE192339) showing the read distribution upstream of the *ylmH* gene in *F. nucleatum*. The p.*rpoE* library shows the read coverage upon induction of σ^E^; ctrl. is the corresponding empty vector control. The TSSs are indicated by an arrow. The sRNA FoxJ is indicated in salmon. (**B**) Genomic synteny of the sRNA FoxJ across different fusobacterial (sub)species (FNN*, F. nucleatum* subsp. *nucleatum*; FNA*, F. nucleatum* subsp. *animalis*; FNP, *F. nucleatum* subsp. *polymorphum*; FNV, *F. nucleatum* subsp. *vincentii*; FuH, *Fusobacterium hwasookii*; and FuP, *Fusobacterium periodonticum*). (**C**) Genomic alignment of representative strains of different fusobacterial species highlighting the strong sequence conservation of FoxJ. The TSSs of FoxJ and *ylmH* are indicated with an arrow. Gray boxes indicate the stop codon of *der* and the start codon of *ylmH*. A dashed line indicates putative Rho-independent terminators of FoxJ. (**D**) Northern blot detection of the sRNAs FoxJ and FoxI using RNA samples of the early (E) and mid-exponential (M) growth phase as well as the stationary phase (S). The 5S rRNA served as a loading control. Both sRNAs were detected on the same membrane, and the same 5S rRNA loading control is shown twice.

Our previous work on the σ^E^ regulon of *F. nucleatum* identified 28 operons with a putative σ^E^-dependent promoter (8). Any previously overlooked σ^E^-dependent sRNA is likely associated with one of these transcriptional units. Our precise definition of the 5′ and 3′ boundaries of transcripts allowed us to search within the UTRs of previously identified σ^E^-activated genes for additional TSSs and TTSs that define short transcripts that might be novel sRNAs. We found that the 5′ UTR of the putative *Bacillus subtilis* homolog *ylmH* harbors a strong internal TTS followed by a weak secondary TSS (Fig. 2A). Moreover, re-analysis of our previous RNA-seq analysis after induction of σ^E^ (8) showed a clear accumulation of reads for the 5′ UTR of the *ylmH* homolog compared to the downstream region (Fig. 2A). We named this putative sRNA FoxJ, following the nomenclature of its companion sRNA FoxI.

A comparison of the genomic region of FoxJ in different fusobacterial species showed a conserved genomic synteny (Fig. 2B). Specifically, the *foxJ* gene is located upstream of *ylmH*, which, in turn, lies between the genes of the 50S ribosomal subunit stability factor, *der*, and the housekeeping 4.5S ncRNA (Fig. 2B). FoxJ displays a high degree of conservation on the primary sequence level (Fig. 2C) but shows no obvious similarity to FoxI (Fig. S2A). To prove that FoxJ indeed accumulates as a discrete RNA species, we performed a northern blot analysis of RNA samples collected from the early and mid-exponential growth phases as well as from the stationary phase. We observed a prominent ~156-nt transcript consistent with the length expected from our 5′ and 3′ end sequencing data (Fig. 2D). The northern blot analysis also shows that FoxJ is expressed throughout all three growth phases with a small decrease during the mid-exponential phase (Fig. 2D). In comparison, FoxI expression increases toward the mid-exponential phase, which indicates a differential regulation between both sRNAs at least under standard growth conditions (Fig. 2D).

### FoxJ expression is σ^E^-dependent

**Fig 3:**
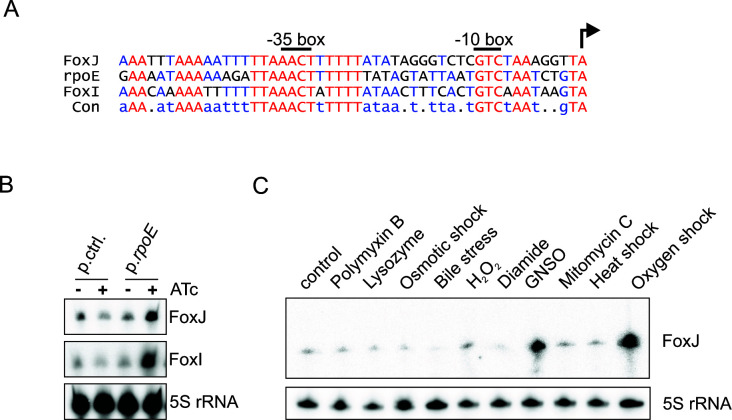
FoxJ as a σ^E^-dependent sRNA. (**A**) Genomic alignment of the promoter region for FoxJ, *rpoE,* and FoxI with the TSS indicated by an arrow. The conserved −10 and −35 boxes are labeled. (**B**) Northern blot detection of the sRNAs FoxJ and FoxI using RNA samples from *F. nucleatum* carrying either a control vector (p.ctrl.) or a vector allowing inducible expression of rpoE (p.*rpoE*). Expression was either induced for 30 min with 100 ng mL^−1^ anhydrotetracycline (ATc) or the samples were left untreated. The 5S rRNA served as a loading control. (**C**) Detection of the FoxJ sRNA via northern blot in total RNA samples extracted from *F. nucleatum* subjected to the indicated stress conditions for a duration of 60 min. The 5S rRNA served as a loading control.

In addition to the primary sRNA sequence, the promoter region of *foxJ* is highly conserved among *F. nucleatum* strains (Fig. 2C). Comparing the promoter regions of *foxI*, *foxJ,* and *rpoE* reveals common −10 and −35 motifs of σ^E^ recognition sites (Fig. 3A). In agreement with our RNA-seq data (Fig. 2A), we also observed a clear accumulation of FoxJ by northern blot after induced ectopic expression of σ^E^ for 30 min (Fig. 3B), in line with previous results for FoxI (8). This dependency on σ^E^ is further confirmed by using a plasmid-based transcriptional reporter assay, in which we placed a 100-bp fragment of the promoter region of *foxJ* upstream of mCherry to drive the transcription of this reporter gene. After the transformation of *F. nucleatum* with this plasmid, we observed a strong fluorescent signal compared to the plasmid-less strain (Fig. S2B). Importantly, the introduction of a point mutation in the σ^E^ motif strongly diminished the reporter activity, supporting the notion that the *foxJ* gene is transcribed by σ^E^ (Fig. S2B).

We recently showed that σ^E^ and FoxI are activated upon oxygen exposure but not by classical envelope stress (8). To assess if FoxJ displays a similar activation pattern, we analyzed FoxJ levels under several stress conditions, including envelope stress (polymyxin B, lysozyme, and bile), osmotic stress (NaCl), oxidative stress (H_2_O_2_, diamide, and S-nitrosoglutathione [GNSO]), DNA damage (mitomycin C), heat shock (42°C), and oxygen exposure. In line with the σ^E^ dependence of FoxJ synthesis, we observed a strong upregulation upon oxygen exposure (Fig. 3C). Exposure to GNSO, which mimics nitrosative oxidative stress, also increased the expression of FoxJ. All other conditions either caused no change or led to a downregulation of FoxJ levels. The transcriptional activation of FoxJ upon oxygen exposure or GNSO treatment closely mimics the activation conditions of FoxI, emphasizing the shared activation of these two sRNA genes. Interestingly, GNSO treatment is unlikely to directly activate σ^E^ in *F. nucleatum* because several σ^E^regulon members are not affected by this stress (8). This suggests that stress induced by GNSO treatment likely depends on an additional transcriptional regulator.

Based on these results, FoxJ is a strong candidate for a second highly conserved sRNA in the noncoding arm of the σ^E^ response in *F. nucleatum*.

### FoxJ acts as a negative regulator of the FoxI-target fomA

**Fig 4:**
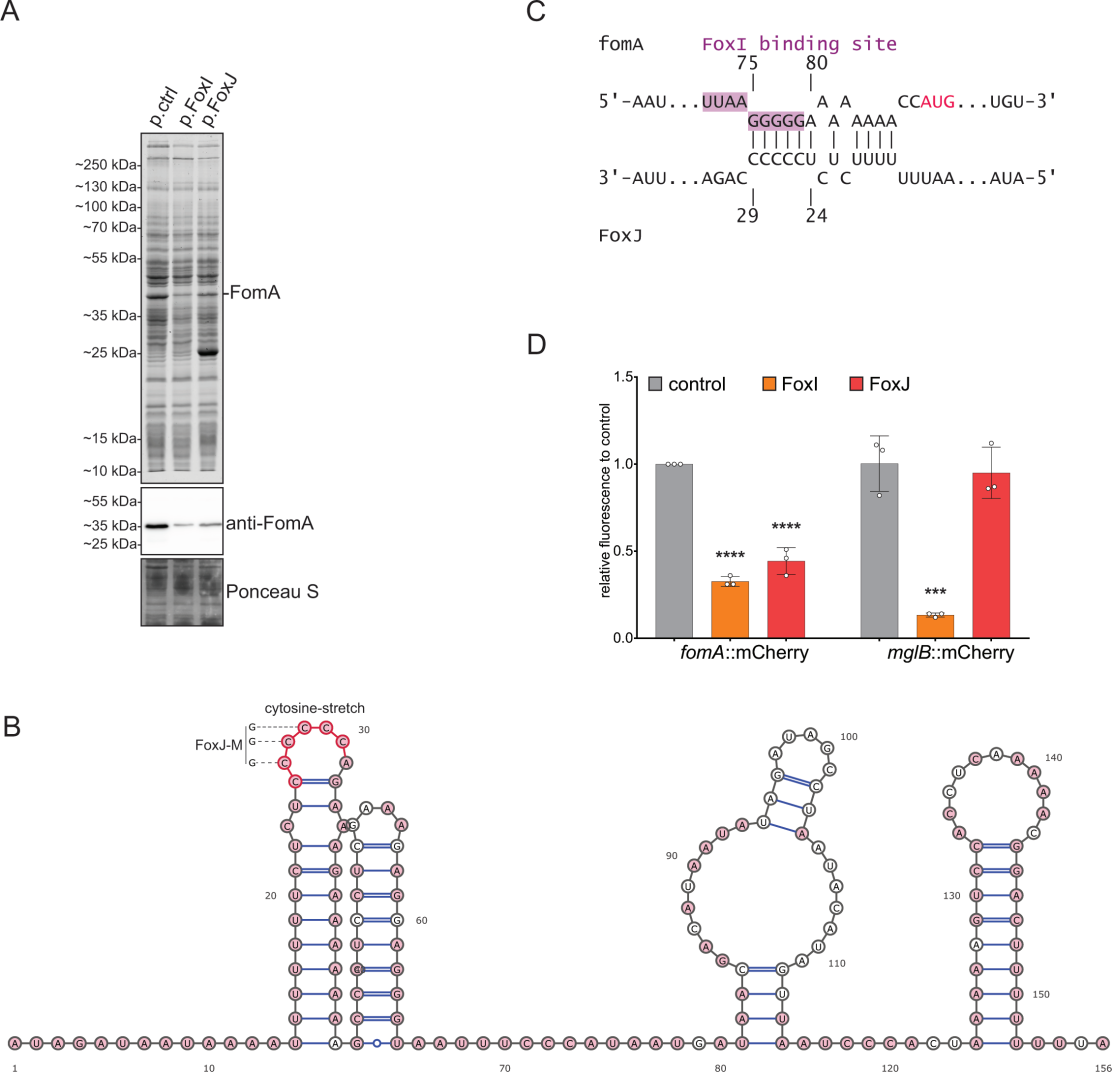
Negative regulation of FomA expression by the sRNA FoxJ. (**A**) (Top) SDS-PAGE analysis visualized via coomassie staining. Equal amounts of OD_600_ units were loaded for *F. nucleatum* carrying the empty control vector (p.ctrl), the FoxI- (p.FoxI), or the FoxJ-overexpressing vector (p.FoxJ). (Middle) Western blot detection of FomA using an anti-FomA antibody. (Bottom) Ponceau S staining served as a loading control for the western blot. (**B**) Secondary structure prediction of FoxJ. Conserved nucleotides (see Fig. 2) are colored in red. The cytosine stretch as likely seed region and the mutations introduced in the FoxJ-M mutant sRNA are indicated. (**C**) *In silico* prediction of the interaction between the FoxJ sRNA and *fomA* mRNA using IntaRNA. The predicted FoxI binding site is highlighted in purple. (**D**) Quantification of the fluorescent signal for the indicated translational fusions with mCherry via flow cytometry. The plasmid carried either an empty expression cassette (control) or that for FoxI (FoxI) or FoxJ (FoxJ) overexpression. The data are displayed as an average and standard deviation of three biological replicates relative to the average of the control (control). Statistical testing was performed using a one-way ANOVA compared to the control group (*P ≤ 0.05; **P ≤ 0.01; ***P ≤ 0.001; and ****P ≤ 0.0001).

As a first step in investigating its targetome, we constitutively overexpressed the FoxJ sRNA from a plasmid. Comparing the total protein profiles of FoxJ expressing (p.FoxJ) to control (p.ctrl) cells by SDS-PAGE, the most prominent change was a strong increase in a ~25 kDa protein (Fig. 4A). Unexpectedly, mass spectrometry analysis of the excised band predicted this protein to be the plasmid-encoded chloramphenicol acetyltransferase (CatP) (Fig. S3A). To clarify this, we added a His-tag to the plasmid-encoded CatP protein. Using an anti-polyHistidine antibody, we again observed a strong FoxJ-induced increase of this protein (Fig. S3B). The reason for the observed accumulation of CatP is unclear, as there is no obvious complementarity between the *catP* mRNA and the FoxJ sRNA; the transcription of these two genes should be independent as well (Fig. S3C). However, we did note a minor increase in relative plasmid DNA upon FoxJ overexpression, which might contribute to the observed accumulation of the CatP protein (Fig. S3D).

More importantly, the overexpression of FoxJ also decreased a prominent band in the 35–55 kDa range, as did the overexpression of FoxI (Fig. 4A). We previously identified this band as the abundant OMP FomA and, subsequently, the *fomA* mRNA as a direct target of FoxI (25). Through western blot analysis with an anti-FomA antibody, we validated the downregulation of this OMP by FoxJ (Fig. 4A), indicating that FoxJ likely shares the *fomA* mRNA as a target with FoxI.

Alignment of available FoxJ sequences revealed a conserved cytosine stretch (Fig. 4B and 2C), akin to the previously identified seed region of FoxI, which is important for base-pairing with the *fomA* mRNA (Fig. 4C) (25). To verify this predicted interaction, we used the previously established *fomA*::mCherry translational reporter system (8) and included an overexpression cassette for FoxJ on the same vector. Like FoxI, FoxJ led to a reduction of mCherry fluorescence compared to the control (Fig. 4D). Thus, both FoxI and FoxJ repress the same target mRNA *fomA*.

FoxI is also known to target the *mglB* mRNA, which is part of the *mglBAC* operon (8). However, in contrast with the shared repression of *fomA*, a *mglB*::mCherry fusion was only regulated by FoxI and not by FoxJ (Fig. 4D). These results indicated that both σ^E^-dependent sRNAs share targets but also have distinct targetomes, similar to the findings for σ^E^-dependent sRNAs in Proteobacteria.

### The global targetome of FoxJ

**Fig 5:**
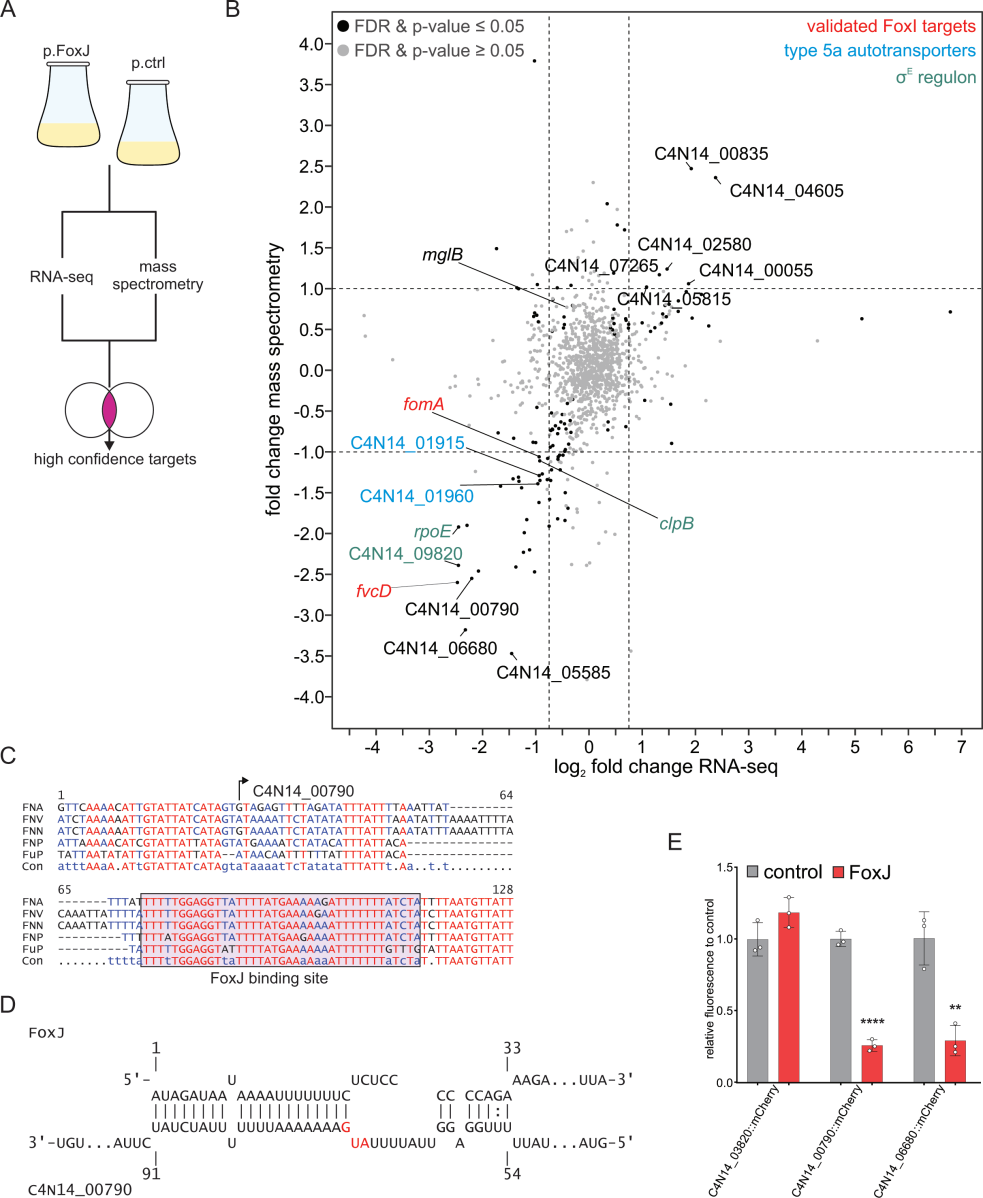
Analysis of the global targetome of the sRNA FoxJ. (**A**) Schematic overview of the workflow for the RNA-seq and mass spectrometry analysis. (**B**) Volcano plot displaying the log_2_ fold changes for all detected genes in both the RNA-seq (*x*-axis; average of 20 raw reads in each group) and fold changes of the mass spectrometry data (*y*-axis; average of label-free quantification ≥ 1×10^6^ in each group). Genes displaying significant gene expression changes (FDR for the RNA-seq data and limma-based *P*-value ≤ 0.05 for the mass spectrometry data) are marked in black. Differentially expressed members of the σ^E^ regulon are marked in green, validated FoxI targets in red, and type 5a autotransporters in blue. (**C**) Genomic alignment of the upstream 5′ region of C4N14_00790 in representative strains of different fusobacterial species. The TSS of the gene is marked with an arrow. The likely binding site of the FoxJ sRNA is indicated. (**D**) *In silico* prediction of the interaction between the FoxJ sRNA and C4N14_00790 mRNA using IntaRNA. The start codon is marked in red. (**E**) Quantification of the fluorescent signal for the indicated translational fusions with mCherry via flow cytometry. The plasmid carried either an empty expression cassette (control) or the FoxJ overexpression cassette (FoxJ). The data represent the average ± standard deviation of three biological replicates relative to the average of the control (control). Statistical testing was performed using an unpaired Student’s *t*-test with Welch’s correction compared to the control group (*P ≤ 0.05; **P ≤ 0.01; ***P ≤ 0.001; and ****P ≤ 0.0001).

To characterize the FoxJ targetome at a global level, we used RNA-seq and mass spectrometry to analyze changes in the *F. nucleatum* transcriptome and proteome, respectively, following overexpression of FoxJ (Fig. 5A). We applied stringent cutoff criteria that require FoxJ to affect both RNA and protein levels of putative targets (RNA-seq cutoff: −0.75 ≤ log_2_ fold change ≥ 0.75; mass spectrometry cutoff: −1 ≤ fold change ≥ 1; both: false-discovery rate [FDR] and *P*-value ≤ 0.05). Thus, we identified 28 downregulated and 6 upregulated genes (Fig. 5B).

Of the 28 downregulated genes, 5 are transport-associated genes, such as a likely *trkA* homolog (C4N14_04580) involved in gating potassium transport (33), three putative genes involved in amino acid transport (C4N14_05105, C4N14_08210, and C4N14_08255) as well as one sugar uptake-related gene (C4N14_03585). An operon encoding the RNase PH, a putative β-ketoacyl-acyl-carrier-protein synthetase (C4N14_03815), and a co-factor synthetase (C4N14_03820) is also downregulated at both the protein and RNA levels. The regulation of this operon combined with the negative regulation of a *fadD* homolog (C4N14_07840) suggests that FoxJ might have a broad impact on fatty-acid metabolism as C4N14_03815 and the *fadD* homolog are likely involved in the initial steps of this metabolic pathway.

We also observed FoxJ-induced downregulation of *fomA,* consistent with the western blot result above. Also, as expected, *mglB* was not among the significantly regulated genes. Besides *fomA*, the FoxI-target *fvcD* was also downregulated by FoxJ (Fig. 5B), indicating that both sRNAs inhibit the expression of type 5 autotransporters encoded by the *fvcD* mRNA. Two additional type 5 autotransporters (C4N14_01915 and C4N14_01960) showed decreased levels (Fig. 5B). Both are encoded by two paralogous operons together with the genes *fadA3a* and *fadA3b*, which are targeted by FoxI (8). This suggests that both FoxI and FoxJ regulate the operon.

The genes most strongly downregulated were C4N14_00790, a putative lipoprotein of unknown function; C4N14_06680, a putative O-antigen modifying enzyme; and C4N14_05585, a hydrolase and methyltransferase domain-containing protein (Fig. 5B). *In silico* target prediction using IntaRNA indicated conserved RNA interactions between FoxJ and the 5′ UTRs of C4N14_00790, C4N14_06680, and C4N14_05585 (Fig. 5C and D; Fig. S4).

To investigate if FoxJ directly regulates the translation of these newly predicted target mRNAs, we generated mCherry translational fusions for C4N14_00790 and C4N14_06680. The C4N14_03820 gene was included as a non-target control. Consistent with the presence of predicted RNA-RNA interactions, FoxJ repressed the C4N14_00790::mCherry and C4N14_06680::mCherry reporters (Fig. 5E) but not the control reporter, C4N14_03820::mCherry.

Taken together, these results show that FoxJ regulates several genes in *F. nucleatum* and likely shares a substantial number of targets with the other σ^E^-induced sRNA, FoxI.

### Positive regulation by FoxJ

**Fig 6:**
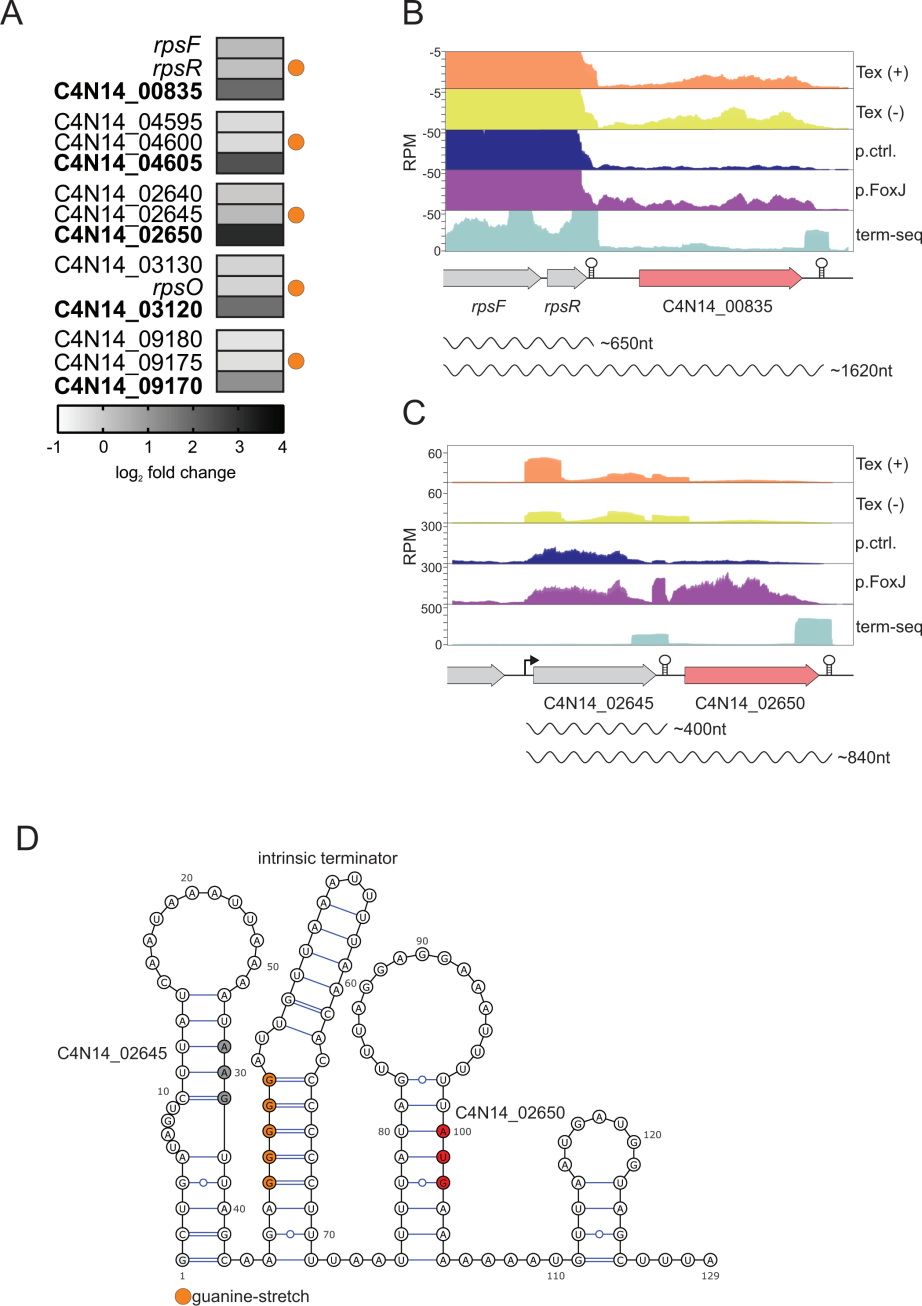
Positive regulation by the sRNA FoxJ. (**A**) Heatmap displaying the log_2_ fold change for genes that are upregulated (bold) upon FoxJ overexpression compared to the control, including the respective upstream genes. Genes that carry a guanine stretch in their 3′ UTR are marked with an orange circle. (**B and C**) Normalized coverage (RPM) from the indicated RNA-seq data showing the read distribution for C4N14_00835 (**B**) and C4N14_02650 (**C**) and the surrounding genomic regions. Annotated TSS and TTS are indicated by an arrow and hairpin symbol, respectively. The lengths of shorter and full-length isoforms are shown below. (**D**) Secondary structure prediction of the intergenic region and the 30 last and first nucleotides for C4N14_02645 and C4N14_02650. The stop codon for C4N14_02645 is marked in gray; the start codon of C4N4_02650 is marked in red. The intrinsic terminator hairpin is labeled, and the guanine stretch is marked with orange circles.

Our combined RNA-seq and mass spectrometry analysis also revealed the upregulation of six genes in both data sets (Fig. 5B). These included the hypothetical gene C4N14_02580; three genes linked to metabolism, *cibK*-like (C4N14_05815), a *ribE* homolog (C4N14_00055), and a *purH* homolog (C4N14_07265), with predicted functions in the biosynthesis of vitamin B12, riboflavin, and purines, respectively; a M48 family peptidase (C4N14_00835); and a putative YwqK family antitoxin (C4N14_04605). In the case of C4N14_02580, C4N14_05815, C4N14_00055, and C4N14_07265, we observed upregulation of their entire respective transcriptional unit (Fig. S5). By contrast, the two top activated genes, encoding the M48 peptidase (C4N14_00835) and a putative YwqK-family antitoxin (C4N14_04605), were the only genes upregulated within their respective operons (Fig. 6A). In both these cases, the genes are the last in a longer operon and neither of them possesses its own TSS (Fig. 6B; Fig. S6A).

The C4N14_00835 gene is located downstream of *rpsR* encoding a 30S ribosome subunit protein (Fig. 6B), while the putative antitoxin lies downstream of another putative antitoxin gene (C4N14_04600) (Fig. S6A). Both these genes are preceded by a putative intrinsic terminator, indicating discoordinate expression of the operons. Manual inspection of the RNA-seq data showed that the overexpression of FoxJ induced a similar discoordinate expression within operons in a total of 14 cases (Fig. 6A; Fig. S6B). A comparison of the intergenic region (IGR) upstream of all positively regulated genes revealed a common guanine-rich stretch in 5/14 cases, which could serve as an interaction site with the cytosine hexamer of FoxJ (Fig. 6A; Fig. S3A). This included the top-upregulated targets C4N14_00835 and C4N14_04605 and three additional targets: C4N14_02650 downstream of C4N14_02645 (Fig. 6C); C4N14_09170 downstream of C4N14_09175; and C4N14_03210 downstream of *rpsO* (Fig. S6C and D). In all cases, our dRNA-seq data did not indicate an independent TSS for the upregulated genes, suggesting a post-transcriptional mechanism involved in their positive regulation (Fig. 6B and C; Fig. S6C and D). Of note, the positive regulation of C4N14_02650, C4N14_09170, and C4N14_03210 was not detected in our global comparative analysis (Fig. 5) due to the cutoffs used for the mass spectrometry data (see Materials and Methods). These putative target sites are located within the intrinsic terminator of the respective upstream genes (Fig. 6D; Fig. S7). To evaluate if this is a common feature in transcriptional terminators of this species, we searched for additional guanine stretches in all identified 3′ UTRs of *F. nucleatum*. We found a similar guanidine-stretch only in 21/663 3′ UTRs. Only the five listed above are positively regulated by FoxJ overexpression (Fig. 6A; Fig. S8). This indicates that this guanine-rich motif is not overly common in fusobacterial 3′ UTRs and that the target specificity is likely influenced by additional factors.

### FoxJ post-transcriptionally promotes mRNA expression of a terminal operon gene

**Fig 7:**
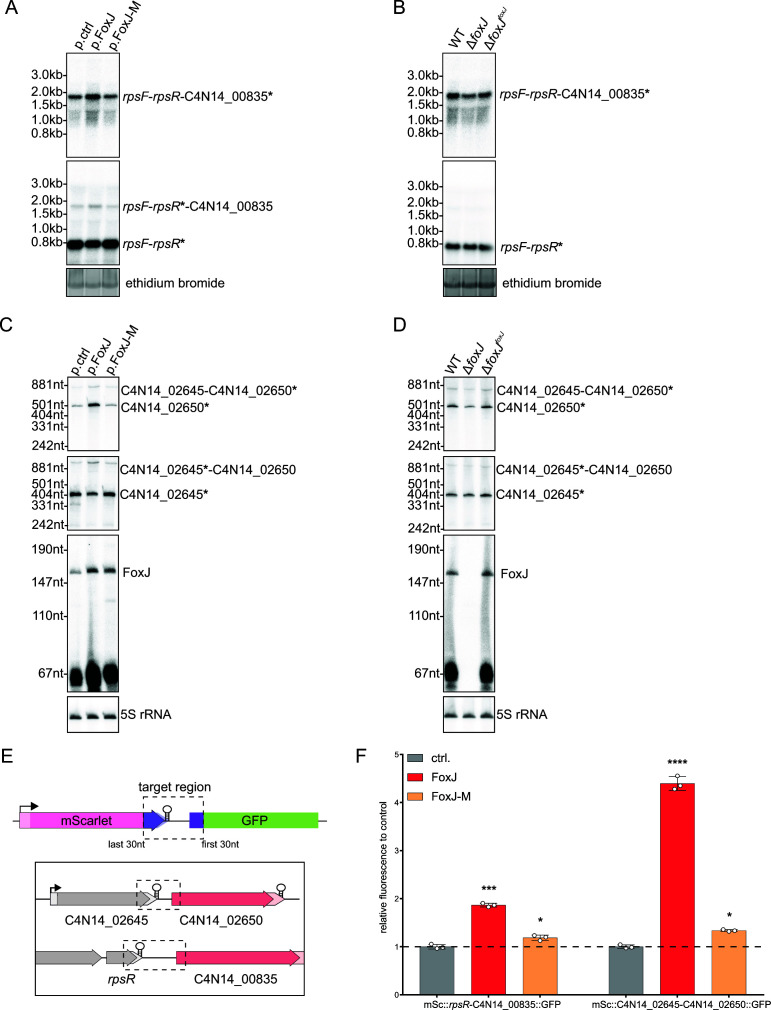
FoxJ post-transcriptionally promotes mRNA expression of terminal operon genes. Northern blot analysis of RNA samples from the mid-exponential growth phase of *F. nucleatum* carrying either the empty vector control (p.ctrl), the FoxJ overexpression vector (p.FoxJ), and that of the seed-region mutant (p.FoxJ-M) (**A and C**) or for samples collected from wild-type *F. nucleatum*, the FoxJ deletion strain (Δ*foxJ*), or the FoxJ-complementation (Δ*foxJ^foxJ^*) (**B and D**). (**A and B**) Detection of the either C4N14_00835 (top) or *rpsR* mRNA (bottom). RNA detected via ethidium bromide staining prior to RNA transfer served as a loading control. (**C and D**) Detection of either the C4N14_02650 (top) or C4N14_02645 mRNA (bottom). 5S rRNA served as a loading control. The asterisk marks the gene that is targeted by the northern blot probe. The quantification for the blots is shown in Fig. S9. (**E**) Schematic representation of the translational reporter system used to investigate the effect of FoxJ on targets at the end of an operon. The dashed regions of the target genes represent the target region placed into the translational fusion vector. (**F**) Quantification of the normalized fluorescent signal for the indicated translational fusions with mSc and GFP via flow cytometry. The plasmid carried either an empty expression cassette (ctrl), that for FoxJ overexpression (FoxJ), or that of the seed region mutant (FoxJ-M). The average of three biological replicates relative to that of the control (ctrl.) is displayed together with the standard deviation. Statistical testing was performed using a one-way ANOVA compared to the control group (ctrl.) (*P ≤ 0.05; **P ≤ 0.01; ***P ≤ 0.001; and ****P ≤ 0.0001).

The positive regulation exerted by FoxJ is specific to the last gene of the target operons, with the respective upstream gene(s) showing no change in expression (Fig. 6A). To further investigate this specific upregulation, we initially focused on the C4N14_00835 gene as one of the top upregulated targets, which displays the largest intergenic distance to its upstream gene (~130 nt). Northern blot probing suggests that C4N14_00835 is most likely part of a polycistronic mRNA, co-transcribed with *rpsF* and *rpsR* (Fig. 7A and 6B). Overexpression of wild-type (WT) FoxJ causes a ~79% upregulation of this multicistronic *rpsF-rspR*-C4N14_00835 mRNA (Fig. 7A; Fig. S9A). Intriguingly, this upregulation was not observed upon overexpression of a FoxJ mutant sRNA, referred to as FoxJ-M (Fig. 7A), which contains a triple C-to-G mutation in the putative interaction site (Fig. 4B).

Next, we generated a FoxJ deletion strain (Δ*foxJ*) to assess the impact of the chromosomally encoded sRNA on the C4N14_00835 mRNA. We observed a ~37% decrease of the *rpsF-rspR*-C4N14_00835 mRNA in the absence of *foxJ*, compared to the WT strain (Fig. 7B; Fig. S9B). Importantly, chromosomal complementation of FoxJ (Δ*foxJ^foxJ^*) fully reversed this effect. These effects were specific to full-length polycistronic mRNA, i.e., when we probed the same blots with a probe directed against the middle gene (*rpsR*), we primarily detected a dicistronic *rpsF-rpsR* on which the overexpression or absence of FoxJ had little if any effect (Fig. 7A and B; Fig. S9E and F). Together, the analysis suggested that it is the synthesis of the full-length *rpsF-rpsR*-C4N14_00835 mRNA that is regulated by FoxJ.

To extend the analysis of FoxJ effects on polycistronic mRNAs, we performed a northern blot analysis of the C4N14_02650 mRNA. Here, we observed two distinct species: a dicistronic C4N14_02645-C4N14_02650 mRNA and a shorter C4N14_02650 mRNA (Fig. 7C). According to our dRNA-seq analysis, the C4N14_02650 gene lacks a TSS (Fig. 6C); therefore, the C4N14_02650 mRNA is likely generated by RNA processing. Overexpression of FoxJ caused a 380% increase of the C4N14_02650 mRNA (Fig. 7C; Fig. S9C), while deletion of the sRNA led to a ~43% decrease (Fig. 7D; Fig. S9D). By comparison, the dicistronic mRNA (C4N14_02645-C4N14_02650) showed a less pronounced increase of ~67% upon overexpression of FoxJ and a 34% decrease in the absence of FoxJ (Fig. 7C and D; Fig. S9C and D). Importantly, the transcript levels of the upstream gene, C4N14_02645, were hardly affected by either overexpression or genomic deletion of FoxJ (Fig. 7C and D; Fig. S9C and D). Thus, as with the C4N14_00835 mRNA, FoxJ primarily acts to upregulate the mRNA of the terminal gene.

### Verification of positive regulation by FoxJ using translational reporters

To study the putative targeting of intra-operonic mRNA sites by FoxJ in a genome-independent manner, we generated a translational fusion reporter system designed to mimic a dicistronic operon, as previously done in *E. coli* (34). The reporter system constitutively expresses mScarlet-I (mSc) and superfolder GFP (GFP) with Golden-Gate compatible restriction sites between the two reporter genes, allowing us to generate 3′ and 5′ translational fusions, respectively (Fig. 7E). We then created translational fusions for the two aforementioned putative FoxJ target regions, i.e., reporters *mSc::rpsR*-C4N14_00835::*GFP* and *mSc*::C4N14_02645-C4N14_02650::*GFP*. Both constructs include the entire intergenic region as well as the last 30 nt of the upstream gene and the first 30 nt of the target gene (Fig. 7E). Fluorescence levels of each construct were determined either alone or upon overexpression of FoxJ, normalizing GFP fluorescence levels to mSc as an internal control. This analysis showed a clear upregulation of the GFP fusions upon overexpression of FoxJ for either target, in line with the initial results of the RNA-seq analysis (Fig. 7F). Importantly, overexpression of the FoxJ-M mutant led to only a minor increase in GFP (Fig. 7F). These results suggest that FoxJ directly base pairs with the terminator region of the leading genes, which causes increased expression of the downstream gene.

## DISCUSSION

**Fig 8:**
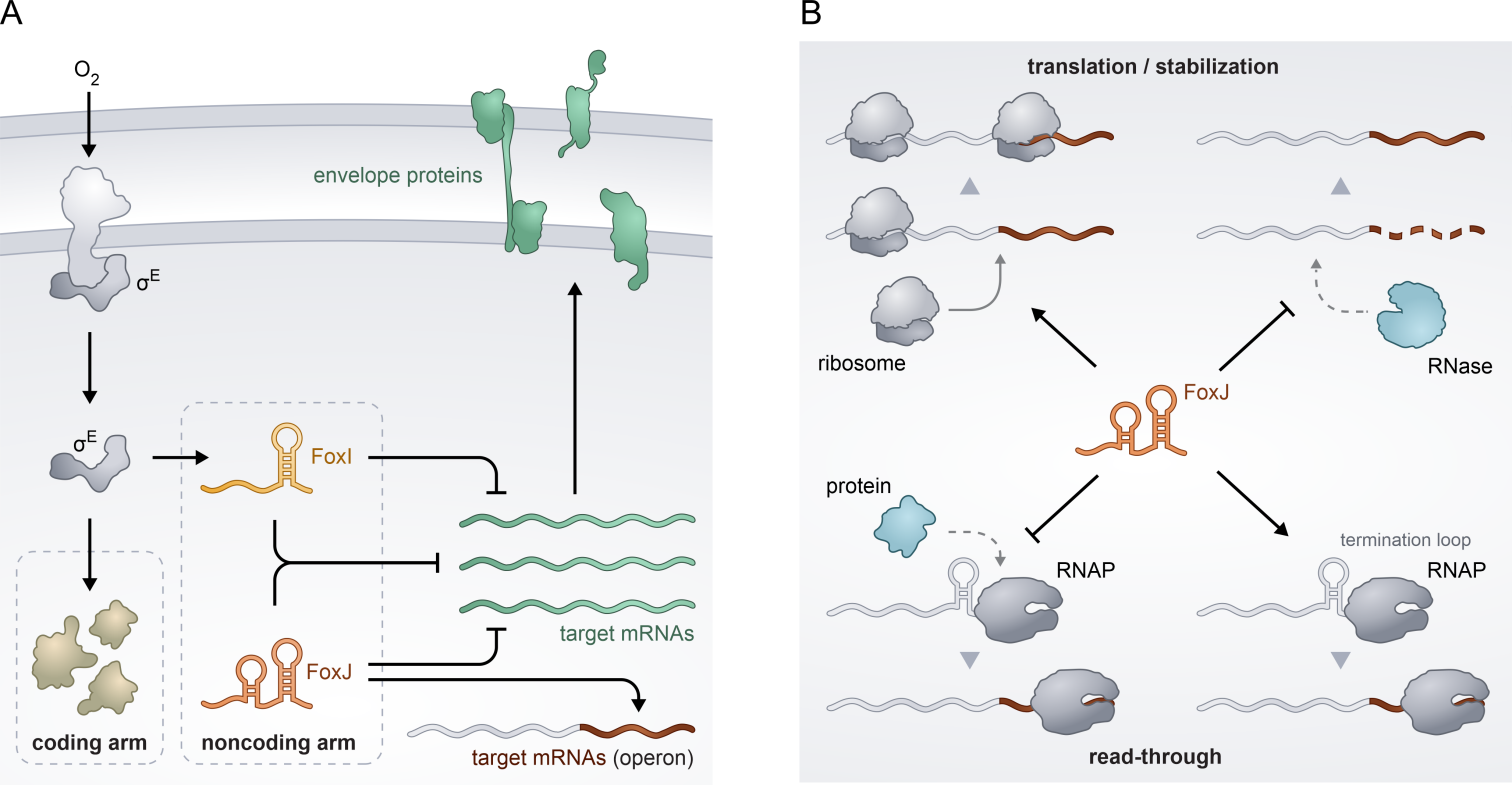
The expanded noncoding arm of the σ^E^ response in *F. nucleatum*. (**A**) Schematic summary of the noncoding arm of the σ^E^-response in *F. nucleatum*. Upon activation of σ^E^, e.g., by oxygen exposure, the sigma factor drives the expression of both sRNA FoxJ and FoxI. Both sRNAs can inhibit the translation of envelope-associated proteins including several shared targets. In addition, the sRNA FoxJ is able to increase the expression of terminal genes in an operon through a yet-undefined mechanism. (**B**) Potential mechanisms through which FoxJ might positively regulate its mRNA targets. FoxJ could inhibit the binding of proteins that promote pre-mature termination of the mRNA or the activity of RNase, an enzyme that destabilizes the full-length transcript. The sRNA might also promote the transcriptional read-through by the RNA polymerase (RNAP), increasing the RNA levels of the full-length transcript. The binding of FoxJ could also increase the translation of the target mRNA, thereby enhancing the stability of the transcript.

The envelope stress response mediated by the ECF σ^E^ is well-characterized in Proteobacteria. We have recently shown that a homologous stress response exists in *F. nucleatum* (8), a species of the evolutionarily distant phylum Fusobacteriota (26). The similarities included the functional conservation of the coding arm as well as the negative regulation exerted by a noncoding arm in the form of the sRNA FoxI (8). Here, we have expanded this noncoding arm by identifying FoxJ as a second σ^E^-dependent sRNA in *F. nucleatum*. We show that FoxJ, like FoxI, functions as a repressor of mRNA translation. Surprisingly, we found that FoxJ also acts as a positive regulator for specific target genes (Fig. 8). As discussed below, the observed patterns are suggestive of a new mechanism of target activation by *trans*-encoded sRNA that warrants a deeper investigation.

### Conserved features of the noncoding sRNA arm of the σ^E^ response in *F. nucleatum*

The noncoding arm of the σ^E^ response in Proteobacteria usually consists of more than one sRNA (14, 17, 35, 36). Our identification of FoxJ as an additional conserved σ^E^-dependent sRNA in *F. nucleatum* shows that this multiplicity is also a feature of this stress response in a far-removed phylum. Another common characteristic of σ^E^-dependent sRNAs in Proteobacteria is that the sRNAs display a shared targetome, such as MicA and RybB in *E. coli*, which both repress the translation of the *tsx* mRNA (14) or VrrA and MicV, which both repress the *ompT* mRNA in *Vibrio cholera* (35). Moreover, MicA/RybB and VrrA/MicV share the abundant OMP OmpA as a target, highlighting their functional conservation across different species (14, 35). Mirroring this aspect of the regulation of the proteobacterial σ^E^-dependent sRNAs, FoxJ and FoxI also exhibit a common targetome including the abundant FomA porin. Although *E. coli* or *V. cholerae* OmpA is not homologous to fusobacterial FomA (amino acid similarity < 19%), the general similarities suggest that the repression of major envelope proteins through the noncoding arm of the σ^E^ response is a highly conserved aspect of the stress response across different phyla.

The repression of OMP synthesis by σ^E^-dependent sRNAs serves as a regulatory negative feedback loop in the envelope stress response in Proteobacteria (14, 19). Damaged or unfolded OMPs trigger the release of σ^E^ from its cognate anti-sigma factor, thus activating the ECF. Therefore, a coordinate dampening of OMP production through the upregulation of σ^E^-dependent sRNAs balances the σ^E^ response. This is apparent upon genetic inactivation of MicA and RybB, which activates the stress response even under normal growth conditions (16, 18, 20, 37). Our results suggest that such a negative feedback loop also exists in *F. nucleatum*. Specifically, 3 out of the 28 genes that are downregulated upon overexpression of FoxJ in *F. nucleatum* belong to the σ^E^-regulon. These include *rpoE* itself, the chaperone *clpB,* and the hypothetical gene C4N14_09820. This is in line with our previous observation that pulse expression of FoxI decreases the expression of *rpoE* and additional regulon members (8). Although we have yet to demonstrate that σ^E^ is induced upon loss of FoxI and/or FoxJ, the fact that overexpression of these σ^E^-dependent sRNAs reduces σ^E^-activity is a feature of the negative feedback loop firmly established in Proteobacteria (13), and our data show that it applies to Fusobacteriota as well.

### Distinct targetome of FoxI and FoxJ despite similar seed regions

Poly-cytosine stretches, such as in FoxJ, are often found in bacterial sRNAs and might represent a hallmark of regulatory RNAs in bacteria with low GC content genomes such as *F. nucleatum* (38, 39). In fact, FoxJ’s companion sRNA FoxI uses a C-stretch to repress the translation of its targets (8, 25). This similarity is reflected in the overlapping targetome of FoxJ and FoxI, best exemplified by the *fomA* mRNA, in which both FoxJ and FoxI likely target the same site. Shared target sites and seed regions can also be found in other sRNAs, for example, in CyaR and RprA, which both target the *hdeD* mRNA in *E. coli* (40), or in GcvB and DapZ, which target the *dppA* and *oppA* mRNA in *Salmonella* (41, 42). Despite their similar binding sites and seed regions, these sRNAs also regulate distinct targets, which we also observe in the case of FoxJ and FoxI. Differences in targetomes are likely caused by differences in or around the seed region, which can contribute to target specificity and efficiency of regulation (43, 44). Another factor that might determine target specificity is the secondary structure of FoxJ and FoxI. According to *in silico* prediction, the cytosine stretch of FoxJ is present in the loop region of a hairpin (Fig. 4B), whereas the one found in FoxI lies in a long linear stretch (25). Other factors might contribute. For example, although *F. nucleatum* lacks common sRNA chaperones such as Hfq or ProQ, it is possible that FoxJ interacts with an RNA-binding protein to facilitate the positive regulation of targets that do not bind to FoxI. We also note that the expression profile differs between both sRNAs, at least under standard growth conditions (Fig. 2D). Our data show that at least one of the σ^E^-dependent sRNAs is expressed in all growth phases, ensuring balanced expression of *fomA* or *fvcD* throughout growth. Regulation of other members of the FoxJ or FoxI targetomes might only be important under certain conditions.

### Divergence of the noncoding arm in *F. nucleatum*

In response to envelope stress, σ^E^ generally functions as an activator of gene expression (45, 46), leading to the transcription of several genes important for OMP biogenesis as well as chaperones and proteases (15, 47, 48). The σ^E^-dependent sRNAs are thought to equip this positive regulator with an essential repressor function required to support membrane homeostasis (13). Importantly, our observation that FoxJ decreases the levels of several mRNAs challenges the concept that σ^E^-dependent sRNAs act solely as repressors. Our discoveries also raise the question if genes activated by FoxJ might be connected to the σ^E^ response in *F. nucleatum*, but finding an answer is challenging because the majority of fusobacterial genes are of unknown function. Nevertheless, one of the targets activated by FoxJ, C4N14_00835, might provide a hint. C4N14_00835 is an M48 peptidase with an N-terminal lipoprotein signaling peptide. Interestingly, the M48 peptidase *ycaL* with a similar signaling peptide is directly activated by σ^E^ in *E. coli*. YcaL works in conjunction with the two proteases BepA and DegP to degrade stalled LptD (lipopolysaccharide assembly protein LptD) on the β-barrel assembly machinery complex and thus contributes to membrane homeostasis (49). Several members of the σ^E^ regulon in *F. nucleatum*, such as *skp* or *bamA*, are likely involved in envelope maintenance or protein translocation as well (8). Thus, it is possible that C4N14_00835 might play a similar role in supporting the quality control of newly synthesized OMPs. In that case, FoxJ would directly activate a gene involved in maintaining envelope homeostasis and thus support the coding arm of the σ^E^ response.

The role of the other genes activated by FoxJ is more elusive because no clear homologs of known functions exist. The C4N14_04605 protein is the only other target that contains a predicted domain, a MORN-domain. Fusobacterial MORN-domain-containing proteins have been suggested to be involved in bacterial adhesion based on their genomic association with autotransporters and other OMPs (50). If and why envelope stress would require a FoxJ-mediated upregulation of a potential adhesin-related protein is currently unclear.

### Discoordinate regulation of operons

A striking observation shared by all five FoxJ-activated genes is that they are the last gene in an operon and that the activity of FoxJ leads to the regulatory uncoupling of the target mRNA from the rest of the operon. Such discoordinate operon expression has been known for the *E. coli* sRNA Spot42, which acts within the *galETKM* operon (51). Specifically, Spot42 inhibits the translation of *galK* but does not affect the other genes of this polycistron (51). More recent examples are provided by the sRNA SdhX, which optimizes carbon flux by translational repression of *ackA* of the *ackA-pta* operon in *E. coli* and *Salmonella* (52, 53). Furthermore, the *Salmonella* NarS sRNA mediates a specific repression of *nirC* as part of the *nirBDC-cysG* operon to balance intracellular nitrite levels (54). In all these examples of sRNA-mediated discoordinate operon expression, the sRNAs fine-tune metabolic pathways and thus help the bacteria to adapt to a changing environment. If such a common function underlies FoxJ-mediated regulation as well remains to be determined; the function of the activated genes is unknown, with the exception of the genes encoding ribosomal 30S subunit proteins in the *rpsF-rpsR*-C4N14_00835 and *rpsO*-C4N14_03120 operons. Further investigation is required to establish whether the observed FoxJ-dependent positive regulation of the terminal genes serves to modulate protein synthesis under σ^E^-inducing conditions.

### A new target activation mechanism for trans-encoded sRNA

While positive target regulation by σ^E^-dependent sRNAs has not been reported before, it is worth considering that multiple other bacterial sRNAs positively affect their mRNA targets directly (55, 56). For example, *Staphylococcus aureus* RNAIII increases target translation by disrupting the formation of an inhibitory secondary structure in the *hla* mRNA (57). In *E. coli*, the sRNAs ArcZ, DsrA, and RprA all activate the translation of the *rpoS* mRNA via the same mechanism (58–61). The RyhB sRNA also activates at least two mRNAs by this mechanism (62, 63). Generally, this “anti-antisense” mechanism appears to be common in diverse bacterial species, such as *Listeria monocytogenes* (64), *Pseudomonas aeruginosa* (65), or *V. cholerae* (66). While we cannot rule out that a similar mechanism contributes to the FoxJ-dependent positive regulation observed here, we consider it unlikely, because such regulation would be expected to require a secondary structure that occludes the ribosome-binding sites in all the different FoxJ targets. We see no evidence for such an intrinsic inhibitory structure.

Another mechanism of sRNA-mediated mRNA activation operates by transcript stabilization. This mechanism was initially found for the sRNA FasX, which stabilizes the mRNA of virulence gene *ska* in group A *Streptococcus* by binding to the 5′ UTR (67, 68). While the exact mechanism of stabilization remains unknown, FasX might act similar to the *Salmonella* sRNA RydC, which stabilizes the *cfa* mRNA by interfering with RNase E-mediated decay (69). Interference with RNase E-mediated mRNA decay is also employed by the *Salmonella* sRNA SgrS. Different from FasX or RydC, SgrS targets the coding sequence of *pglB* of the *pglB-yigL* operon, which leads to a selective stabilization of a processed *yigL* mRNA (70). Of the five upregulated targets by FoxJ, only the C4N14_02645-C4N14_02650 mRNA shows evidence of RNA processing (Fig. 6C and 7C). However, binding of FoxJ to the mRNA increases the levels of both the full-length and shorter transcript, suggesting that the sRNA is not directly involved in the processing of the operon.

Regulatory sRNAs may also interfere with the binding of proteins other than RNases to activate target mRNAs. For example, sRNAs can inhibit the activity of the transcriptional terminator Rho to increase target mRNA synthesis. This has been proposed for the sRNA SraL, which binds to the 5′ UTR of the *rho* mRNA in *Salmonella*. This interaction interferes with the binding of the Rho protein to its own transcript, thus reducing Rho-mediated termination and subsequently increasing the levels of the full-length mRNA (71). Regulation by anti-termination was also shown to be involved in the aforementioned *rpoS* mRNA activation by ArcZ, DsrA, and RprA (72). Here, the sRNA-mRNA interaction occurs during transcription to mask Rho-specific binding sites referred to as “rut sites” or to block Rho translocation along the mRNA. Both are required for Rho-dependent termination (73, 74).

The genome of *F. nucleatum* encodes a Rho homolog, and it is therefore possible that FoxJ might interfere with Rho-dependent termination. However, the putative interaction sites of FoxJ on the positively regulated mRNAs share a common GC-rich palindrome followed by a uridine track. These features represent all characteristic structural elements of an intrinsic terminator. Intrinsic termination via the formation of hairpin-like structures destabilizes the RNA polymerase elongation complex in a protein-factor-independent manner (31). Therefore, we currently favor a mechanistic model in which the binding of FoxJ interferes with the formation of or directly destabilizes intra-operonic terminator hairpins, thus counteracting transcription termination. The increased read-through of the RNA polymerase would raise mRNA levels of the downstream gene. While this hypothesis still needs to be thoroughly tested in a reconstituted *in vitro* system to exclude confounding factors such as ongoing translation or binding of unknown cellular proteins, it would constitute a novel co-transcriptional mechanism of regulation. In light of the early divergence of Fusobacteriota (26), it is possible that this mode of action was acquired by *F. nucleatum* specifically or that it has been lost by other phyla after the evolutionary separation. However, since most of our understanding of sRNA-mediated post-transcriptional gene regulation is derived from a limited number of model microbes, it is possible that sRNAs that target terminator hairpins are also found in other species. Proving this putative mode of action of mRNA activation by targeting intra-operonic terminators would be another great example of how the study of phylogenetically distant non-model species may help shed light on the full mechanistic diversity of RNA-based regulation in bacteria.

## MATERIALS AND METHODS

### Strains and growth conditions

All strains, plasmids, and oligonucleotides used in this study can be found in Table S1E. *Fusobacterium nucleatum* subspecies *nucleatum* ATCC 23726 (*F. nucleatum*) was procured from the American Type Culture Collection (ATCC). Cultivation of *F. nucleatum* was routinely conducted at 37°C in an 80:10:10 atmosphere of N_2_, H_2_, and CO_2_ on plates with brain–heart infusion (BHI) broth and 2% agar (BHI-C). The BHI-C plates were composed of BHI, 1% (wt:vol) yeast extract, 1% (wt:vol) glucose, 5  µg mL^−1^ hemin, and 1% (vol:vol) fetal bovine serum. Growth in liquid culture was performed in Columbia broth. Plasmid maintenance was ensured by supplementing BHI-C agar plates or Columbia broth with 5 or 2.5 µg mL^−1^ thiamphenicol, respectively. To maintain anaerobic conditions, all solutions or plates were reduced overnight in the anaerobic chamber to eliminate entrapped oxygen. Pre-cultures of *F. nucleatum* were prepared 24 h prior to inoculating the working cultures at a 1:50 dilution.

### Construction of gene deletion system pVoPo-06

Based on pVoPo-04 (8), we generated an improved deletion vector to allow the insertion of required homologous regions via Golden Gate cloning including a GFP-dropout control. The initial vector was opened via inverse PCR (JVO-18369/JVO-18370). The open vector was assembled together with JVO-22111 using the NEBuilder Hifi Assembly Cloning kit (New England Biolabs) to insert a multiple-cloning site with two Esp3I and two PaqCI sites. Next, a constitutive sfGFP expression cassette (*E. coli* codon usage, pFP518) was amplified and inserted into the PaqCI sites of the vector. This vector is called pVoPo-06. The required regions of homology for gene deletion or insertion can be inserted into the Esp3I sites using the Golden Gate cloning strategy. The green fluorescent signal of GFP can be used to discard negative colonies in the screening process for this cloning step.

### Construction of translational fusions for studying the post-transcriptional regulation

The pVoPo-02 system (8) was used as the backbone. The FoxJ or FoxJ-M overexpression cassette was inserted into the EcoRI site. The target region, containing the 5′ UTR and the first 30 nucleotides of the target gene, was placed into the ScaI and XhoI sites of the vector to yield in-frame translational fusions with mCherry.

To study the post-translational regulation of genes that are part of an operon, we generated pVoPo-07. We used pFP217 as a backbone. This vector constitutively expresses mScarlet-I from the fusobacterial *accA* promoter, also used for pVoPo-02. The vector was opened via inverse PCR to contain terminal NheI and XhoI sites. sfGFP was amplified from pVoPo-GFP with similar restriction sites and ligated into the opened vector, yielding a vector expressing an mSc-GFP dicistronic mRNA. This vector was opened again via inverse PCR, removing the IGR between both genes including the stop codon of mSc and the start codon of GFP. This product was assembled together with the oligo JVO-22079 inserting two BsaI sites between both genes. To generate translational fusions of interest, we amplified products containing the last 30 nucleotides up to the first 30 nucleotides of the up- and downstream genes, containing matching BsaI sites on both ends. We then ligated the BsaI-digested vector and the amplified target region to yield the desired translational fusions (for schematic, see Fig. 7).

### Transformation and gene deletion in *F. nucleatum*

Preparation of electro-competent *F. nucleatum* and subsequent transformation were conducted as previously described (8, 25). In the case of replicative plasmids, ~200 ng was transformed into ~5 OD of cells in 10% (vol/vol) glycerol. The amount of plasmid was increased to ~5 µg for suicide plasmids and included a de-salting step (>6 h) of the DNA prior to the transformation. In the case of generating gene deletions, successful first integration events, based upon selection on thiamphenicol plates, were plated on anhydrotetracycline (ATc) containing plates (200 ng mL^−1^) after a working culture was induced for 4 h with 100 ng mL^−1^ ATc. Plasmid loss was verified by re-streaking colonies on BHI-C plates with or without thiamphenicol followed by PCR verification.

### Detection of RNA via northern blot

In order to detect RNA, we either conducted northern blot analysis on 6% polyacrylamide gels (PAA) as described before (25) or on 1.2% (wt/vol) agarose gels containing 1% formaldehyde following published protocols (54). Five micrograms of total DNase I-digested RNA was used in the case of PAA gels, while 20 µg was used for agarose blots. In both cases, RNA was transferred to Hybond-N^+^ membranes (GE Healthcare) using electro-blotting (PAA gels) or capillary blotting (agarose gels). The hybridized membranes were incubated with gene-specific [γ^32^]-ATP end-labeled deoxyribonucleotide probes (Table S1E). The radioactive signal was detected by exposing phosphor screens (Fuijifilm) to the membranes followed by the detection via a Typhoon FLA 7000 phosphoimager (GE Healthcare). Quantification was carried out using ImageJ (75).

### Protein detection via coomassie staining and western blot

Protein samples were collected from bacterial cultures in mid-exponential growth, snap frozen, and resuspended in protein loading buffer. Equal OD_600_ units were loaded on denaturing SDS-polyacrylamide gel for SDS-PAGE analysis. To visualize proteins, the gels were stained with Coomassie (Quick Coomassie Stain, Neo Biotech) and destained in double distilled H_2_O (ddH_2_O). For western blotting, proteins were transferred to polyvinylidene fluoride membranes. Prior to blocking, we performed Ponceau S staining to ensure equal loading of the samples. FomA levels were detected using an anti-FomA antibody (25) in combination with an anti-rabbit secondary antibody (Thermo Fisher Scientific, catalog no. 31460). The polyHis-tag was detected using a monoclonal anti-polyHis antibody (Sigma, H1029) in combination with an anti-mouse secondary antibody (Thermo Fisher Scientific, catalog no. 31450). Quantification was carried out using ImageJ (75).

### RNA sample collection

In all experiments, three biological replicates from independent pre-cultures were collected from the mid-exponential phase for each sample and the indicated strains. The total RNA was extracted following the “hot phenol” extraction protocol as previously described (25). Briefly, bacterial pellets were resuspended in lysis buffer (600 µL of 0.5 mg mL^−1^ of lysozyme in Tris–ethylenediaminetetraacetic acid buffer, pH 8.0 with 60 µL 10% sodium dodecyl sulfate) and incubated at 65°C for 2 min. The lysis reaction was stopped through the addition of 65 µL of 3 M sodium acetate (pH 5.2). Next, 700 µL phenol was added, and the samples were incubated at 65°C for 6 min. This was followed by a centrifugation step (13,000 rpm at 4°C for 10 min) followed by the isolation of the aqueous phase. The aqueous phase was subjected to another round of phase separation through centrifugation after the addition of 750 µL of chloroform. The subsequently isolated aqueous phase was precipitated overnight in a 30:1 mix (ethanol: 3 M sodium acetate [pH 6.5]). The next day, the precipitated nucleic acids were pelleted (13,000 rpm at 4°C for 30 min), washed once with 75% (vol/vol) ethanol, and dried prior to re-suspending it in diethyl pyrocarbonate-treated H_2_O. These samples were further freed of DNA by performing a DNase digestion step. The purification of the final DNase-free RNA was achieved by another step of phase separation and precipitation.

### NanoLC-MS/MS analysis of protein samples

The NanoLC-MS/MS analysis was carried out at the technology platform mass spectrometry of the Rudolf Virchow Center for mass spectrometric analyses. Quantification of the single upregulated protein band upon FoxJ overexpression (Fig. 4) was done following the procedure described in reference 25. After separation on a denaturing SDS gel, the band of interest was excised for independent biological duplicates. After destaining in a solution of 100 mM ammonium bicarbonate containing 30% acetonitrile, the samples were then dehydrated in 100% acetonitrile prior to trypsin digestion (using 0.1 µg trypsin in 100 mM ammonium bicarbonate, overnight at 37°C). The samples were diluted in 5% formic acid. In the case of total protein analysis (Fig. 5), the samples were reduced in 50 mM DTT for 10 min at 70°C and subsequently alkylated with 120 mM iodoacetamide for 20 min at room temperature in the dark. Following this, the proteins were precipitated using acetone overnight at −20°C. The precipitate was washed with acetone and then dissolved in 50 µL of 8 M urea and 100 mM ammonium bicarbonate. Protein digestion into peptides was carried out using Lys-C (Wako) for 2 h at 30°C, followed by overnight digestion with trypsin. The resulting peptides were eluted with 60% acetonitrile/0.3% formic acid and stored at −20°C until LC-MS/MS analysis.

In both cases, measurements were carried out on an Orbitrap Fusion ETD (Thermo Scientific) equipped with a PicoView Ion Source (New Objective) and a nEASY-LC1000 liquid chromatography system (Thermo Scientific). The resulting data were analyzed using MaxQuant (v.1.5.7.4) with integrated Andromeda comparing it against the Uniprot database for *F. nucleatum* subsp. *nucleatum* ATCC 23726 merged with that of the plasmid pFP421 (p.FoxJ). For the analysis of Fig. 5, all proteins were required to average label-free quantification values ≥ 1 × 10^6^ for either the control samples or those of the FoxJ overexpression in order to be considered detected.

### Sample collection and analysis for transcriptional and translational reporter experiments

Individual reporter constructs were constructed as previously described (8). For the transcriptional reporters, the 100-bp promoter region of the gene of interest was inserted into pVoPo-01 to drive the expression of mCherry as a reporter protein. Translational fusions were constructed as described above. Samples of *F. nucleatum* carrying the individual transcriptional or translational reporters were grown to the mid-exponential phase, spun down, and fixated for 30 min at 4°C in 4% (wt/vol) paraformaldehyde. Afterward, the samples were washed 2× in phosphate-buffered saline (PBS) and stored overnight at 4°C to allow the maturation of the fluorescent protein. The next day, the samples were stained with 4′,6-diamidino-2-phenylindole (DAPI) in PBS for 5 min. Following one wash in PBS, fluorescence intensity was measured via flow cytometry at 525–545 nm (GFP) and 615–620 nm (mCherry/mScarlet-I) for 50,000 DAPI^+^ cells.

### cDNA library preparation for total RNA-seq

The cDNA library preparation and sequencing for the total RNA-seq analysis were carried out by the Core Unit Systems Medicine (Core Unit SysMed) of the Medical Faculty of the University of Würzburg and the Interdisciplinary Center for Clinical Research of the University Hospital Würzburg. First, ribosomal RNA was removed using RiboCOP META depletion kit followed by ultrasound treatment (one pulse of 30 s at 4°C). An adapter was then ligated to the 3′ end of the fragmented RNA molecules. For the synthesis of first-strand cDNA, the M-MLV reverse transcriptase was used; the introduced 3′-adapter served as a primer. The 5′ Illumina TruSeq sequencing adapters were ligated to the cDNA, and the cDNA was PCR amplified (10–20 ng µL^−1^). The amplified cDNA was purified using the Agencourt AMPure XP kit (Beckman Coulter Genomics) and subsequently assessed via capillary electrophoresis. The cDNA was pooled and further purified through preparative agarose gel electrophoresis leading to fragments of cDNA ranging from 200 to 600 bp. The resulting pooled libraries were sequenced using an Illumina NextSeq 2000 system with 100 bp read length.

### cDNA library preparation for dRNA-seq and term-seq

The cDNA library preparation was carried out by Vertis Biotechnology AG. Generation of the dRNA-libraries was performed as previously described (25). The term-seq libraries were generated by first ligating the 5′ TruSeq Illumina adapter to 3′ hydroxyl ends of rRNA-depleted RNA via a probe-based approach. This RNA was used as input for first-strand cDNA synthesis using M-MLV reverse transcriptase. The cDNA was fragmented via ultrasound followed by the ligation of the 3′ TruSeq Illumina adapter. cDNA was PCR amplified with a high-fidelity DNA polymerase and purified with Agencourt AMPure XP beads (catalog number: A63881; Beckman Coulter Genomics). The resulting libraries were analyzed via capillary electrophoresis. Sequencing of dRNA-seq and term-seq libraries was performed by the Core Unit SysMed using an Illumina NextSeq 2000 system with 100 bp read length.

### Read mapping and analysis of RNA-seq

The FASTX toolkit (v.0.10.1) was used for trimming and filtering of reads. READemption (v.2.0.1) (http://hannonlab.cshl.edu/fastx_toolkit) was used for mapping the reads against the genome sequence of *F. nucleatum* subsp. *nucleatum* ATCC 23726 (NZ_CP028109.1) downloaded from the National Center for Biotechnology Information. Differential gene expression analysis for the total RNA-seq (empty vector control vs FoxJ overexpression) was performed using DEseq2 (v.1.40.2) (76). Genes that showed less than 20 raw reads as a group average were not considered. Differentially expressed genes were defined as having a log_2_ fold change of ≤−0.75 or ≥0.75 with a false-discovery rate of ≤0.05. The dRNA-seq were analyzed using ANNOgesic (v1.0.0) (77) to annotate TSS and 5′ UTRs as previously described (25). The annotation of TTS and 3′ UTRs was conducted manually using the term-seq data. Normalized coverage files were visualized using Integrative Genomics Viewer and scanned for enriched regions. The TTS were annotated with its first position displaying <50% of the maximal read count for the enriched region observed in term-seq data.

### *In silico* target prediction

IntaRNA (v3.3.2) (78) was used to predict sRNA-mRNA interactions. We used the complete 5′ UTR including the first 50 nt of the coding sequence as input for the target sequence. In the case of targets within an operon, we included the entire intergenic region as well as the last 30 nt of the upstream gene and the first 30 nt of the target gene for the prediction. The prediction was performed using the default settings but running it in the heuristic mode and allowing a seed region of only three base pairs.

### Analysis of relative plasmid DNA amount

*F. nucleatum* carrying either the p.ctrl or the p.FoxJ plasmid were grown to the mid-exponential phase. Total DNA was extracted from equal OD_600_ units by pelleting the sample and washing it in ddH_2_O prior to boiling the samples in ddH_2_O for 5 min at 95°C. Next, the samples were vortexed to further disrupt the cell envelope. Following a 5-min centrifugation step at 13,000 rpm, the supernatant was mixed with 1 volume of chloroform through vortexing for 30 s. This was followed by another centrifugation step (13,000 rpm for 10 min at 4°C) after which equal amounts of the aqueous phase were taken for each sample. To evaluate the relative plasmid amounts between both groups, we performed quantitative PCR normalization using the Takyon No ROX SYBR 2× Mastermix (Eurogentec; UF-NSMT-B0701). The quantification was carried out by applying the 2^-ΔΔCt^ method, using the *fomA* gene as a reference gene for normalization.

### Statistical analysis

We applied an unpaired Student’s *t* test with Welch’s correction when comparing only two groups. For more than two groups, we performed a two-way analysis of variance with Dunnett’s correction. In all cases, a *P*-value ≤ 0.05 was considered a significant change and further marked as follows: *, P-value ≤ 0.05; **, *P*-value ≤ 0.01; ***, *P*-value ≤ 0.001; and ****, *P*-value ≤ 0.0001. We performed all experiments as three independent biological experiments, which were used for the statistical analysis.

### Data visualization

Coverage plots were generated with the R packages Gviz (v.1.32.0). RNA secondary structures were predicted by Mfold (79) and visualized with VARNA (80).

## Data Availability

RNA-seq data can be accessed at NCBI’s GEO under GSE249955. MS data can be accessed at the Proteomics Identification Database PRIDE under PXD047655.

## SUPPLEMENTAL MATERIAL

Supplemental Figures mbio.03536-23-s0001.pdf Fig. S1-S9.

Supplemental Table mbio.03536-23-s0002.xlsx Table S1A-E.

ASM does not own the copyrights to Supplemental Material that may be linked to, or accessed through, an article. The authors have granted ASM a non-exclusive, world-wide license to publish the Supplemental Material files. Please contact the corresponding author directly for reuse.
