# Supplementary material for: Transcriptome fine-mapping in Fusobacterium nucleatum reveals FoxJ, a new σE-dependent small RNA with unusual mRNA activation activity
Source: mBio. 2024 Mar 4;15(4):e03536-23. doi: 10.1128/mbio.03536-23 (PMC11005410; doi:10.1128/mbio.03536-23)
Supplement: Supplemental Figures — Fig. S1-S9. [file mbio.03536-23-s0001.pdf]

Supplementary Fig. S1

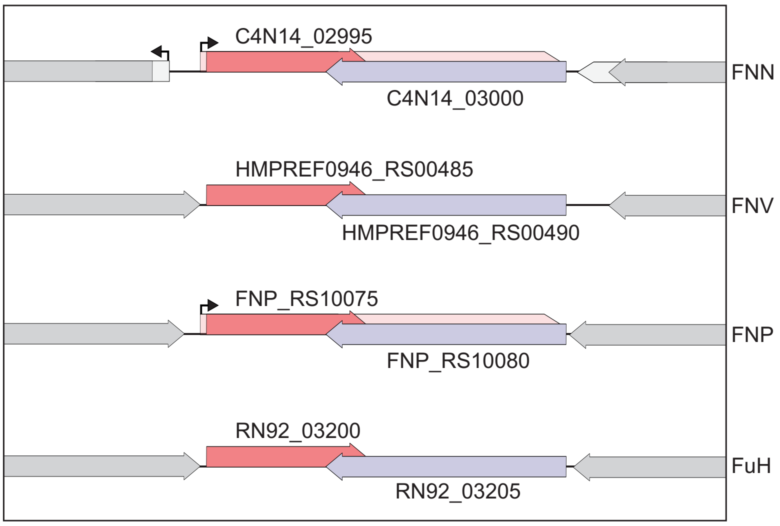

Fig. S1 A conserved antisense configuration for C4N14_02995 and C4N14_03000.

Schematic representation of the genomic arrangement for C4N14_02995 and C4N14_03000 across representative strains of different fusobacterial species.

Supplementary Fig. S2

A

```
      1                                                                            78
FoxJ  ATAGATAATAAAATTTTTTTCTCTCCCCCCAGAAAGAAAAAAAGCCCTCCTCGAAAGAGGAGGGTAATTTCCCATAAT
FoxI  ATACAGATAAAATT---------------------GATAAAATACCCCCTAACAATGAGCAGTATGATATC-------
 Con  ATACAgAaaAAAaT.....................GAaAAAAaaCCCcCcaacAAaGAGcAGgaTaATaTC.......

      79                                                                          156
FoxJ  GATAACGACATAATATAGATAGCCTAATACATAGTTAATCCCACTAAAAAGTCCACCTCAAAAACGGACTTTTTTTTA
FoxI  GAAAATACGATAATTATAACTGCTCATTTTATTTTTT
 Con  GAaAAcaacATAATaaaaAcaGCccAaTacATagTTa.........................................
```

B

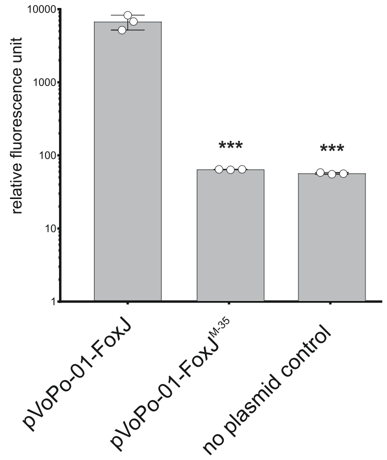

Fig. S2 Comparison of FoxJ to FoxI and σ^E-dependency.

(A) Genomic alignment of FoxJ and FoxI of *F. nucleatum* subsp. *nucleatum* ATCC 23726 (Con, consensus sequence). (B) Quantification of the fluorescent signal for mCherry for the transcriptional reporters encoding either the native promoter of FoxJ (pVoPo-01-FoxJ), one carrying a single point mutation in the -35 region of the FoxJ promoter (pVoPo-01-FoxJ^{M-35}) or for a no plasmid as background control. The average of three biological replicates relative to thhe average of the control (control) is displayed together with the standard deviation. Statistical testing was performed using a one-way ANOVA compared to the control group (ctrl.) (*: P ≤ 0.05; **: P ≤ 0.01; ***: P ≤ 0.001; ****: P ≤ 0.0001).

Supplementary Fig. S3

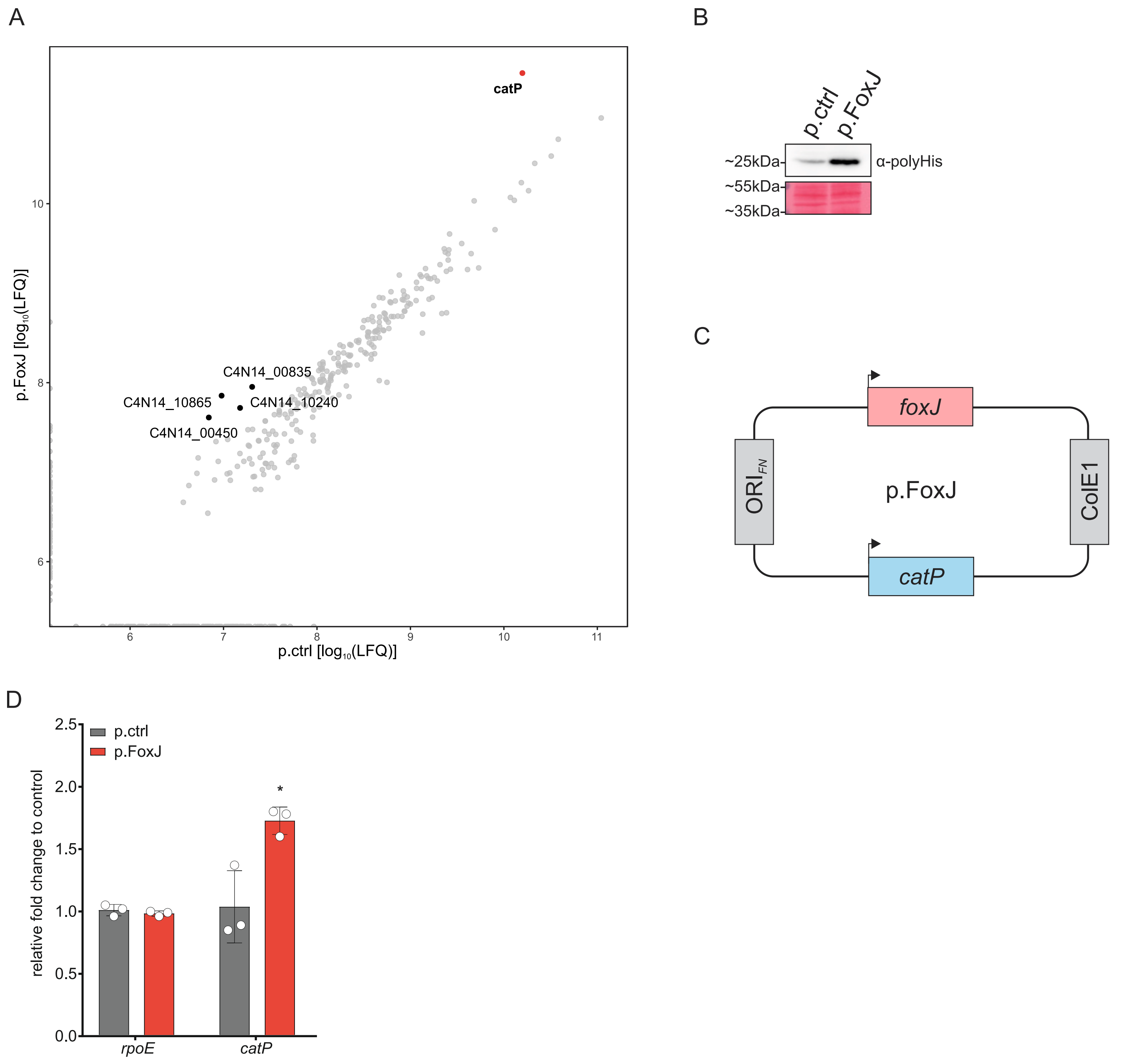

Fig. S3 Indirect positive regulation of *catP* expression by FoxJ.

(A) LC–MS/MS analysis comparing the label-free quantification (LFQ) values of samples overexpressing FoxJ to the empty vector control. (B) Western blot analysis for protein samples of *F. nucleatum* carrying either the empty vector control (p.ctrl) or the FoxJ overexpression vector (p.FoxJ) with a C-terminally his-tagged *catP* gene (*catP*::8xHis). Ponceau S staining served as a loading control. (C) Schematic representation of the plasmid containing the FoxJ overexpression cassette. The empty vector control lacks FoxJ but is otherwise identical. (D) Results of the quantitative PCR for rpoE and catP using total DNA samples harboring either the empty vector control or the FoxJ overexpression vector. The results were normalized to the abundance of the *fomA* gene. The relative amounts of the *rpoE* gene were selected as representative for the relative amounts of genomic DNA, whereas *catP* DNA was measured to evaluate the relative amount of plasmid DNA compared to the genomic DNA in the sample. The average of three biological replicates relative to that of the control (p.ctrl) is displayed together with the standard deviation. Statistical testing was performed using an unpaired Student's t-test with Welch's correction compared to the control group (p.ctrl.) (*: $P \leq 0.05$; **: $P \leq 0.01$; ***: $P \leq 0.001$; ****: $P \leq 0.0001$).

Supplementary Fig. S4

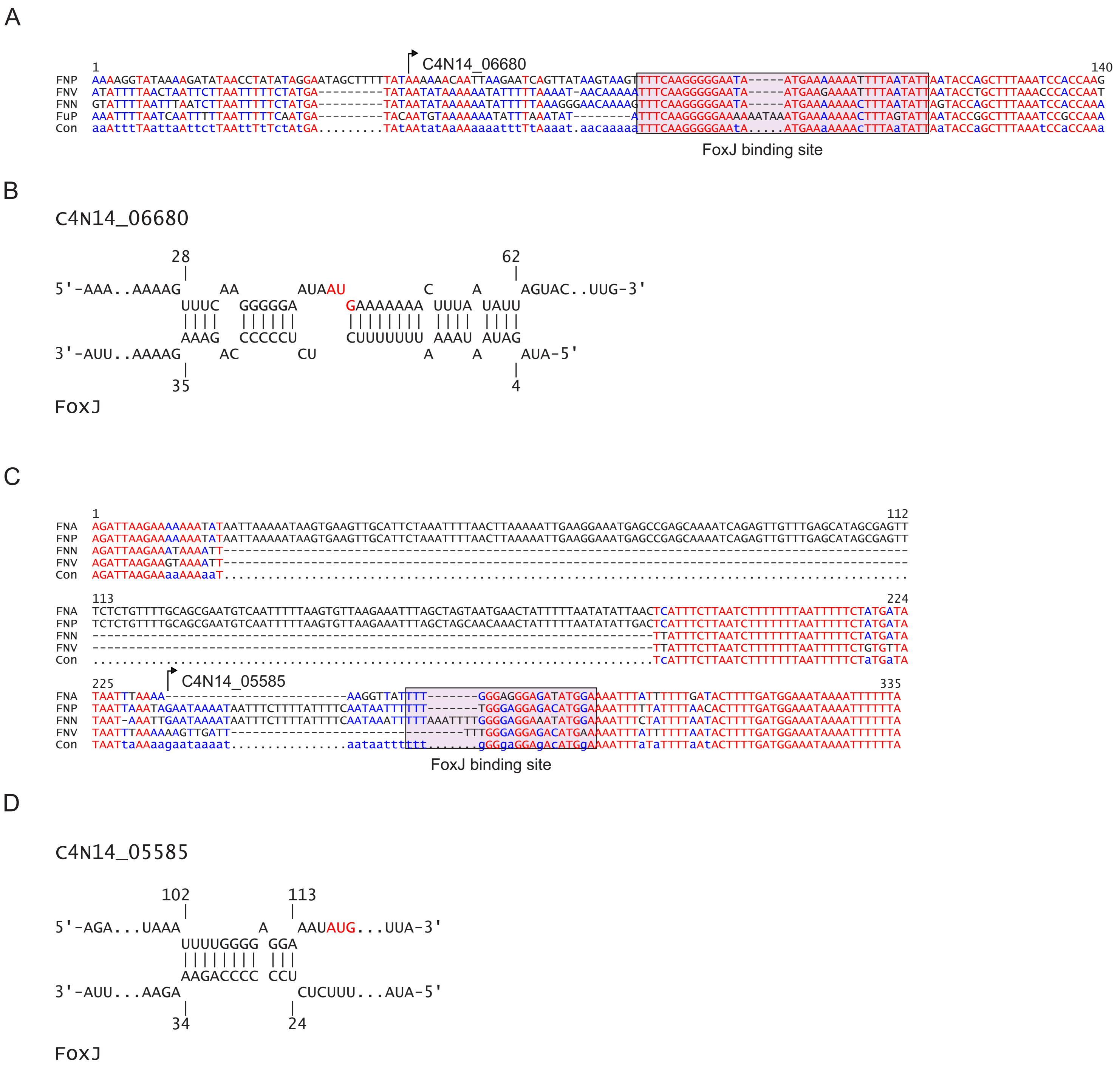

Fig. S4 Overview for additional targets of FoxJ.

(A) Genomic alignment of the 5' region of C4N14_06680. (B) *In silico* prediction of the interaction between the FoxJ sRNA and C4N14_06680 mRNA using IntaRNA. (C) Genomic alignment of the 5' region of C4N14_05585. (D) *In silico* prediction of the interaction between the FoxJ sRNA and C4N14_05585 mRNA using IntaRNA. TSS are indicated by arrows. The putative FoxJ binding site is highlighted by boxes. FNN*, F. nucleatum* subsp. *nucleatum*; FNA*, F. nucleatum* subsp. *animalis*; FNP, *F. nucleatum* subsp. *polymorphum*; FNV, *F. nucleatum* subsp. *vincentii*; Con, consensus sequence.

Supplementary Fig. S5

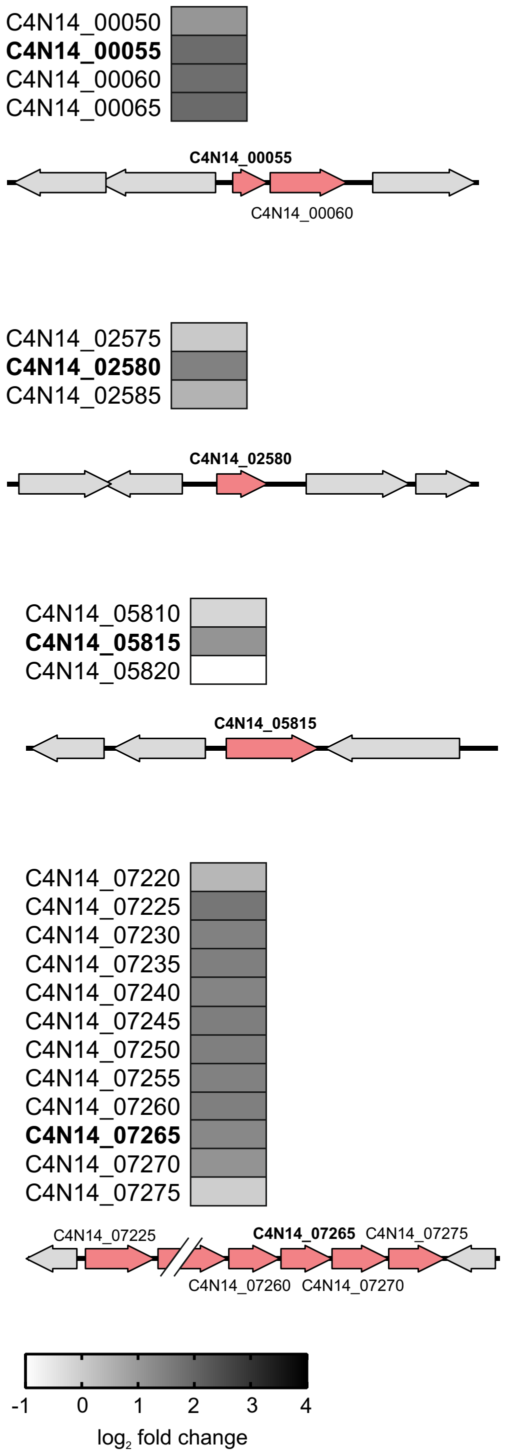

Fig. S5 Additional targets that are upregulated upon overexpression of FoxJ.

Heatmaps displaying the log_2 fold changes for the gene expression upon FoxJ overexpression compared to the control. The corresponding representation of the genomic organization for additional targets that are upregulated upon FoxJ overexpression are also shown.

Supplementary Fig. S6

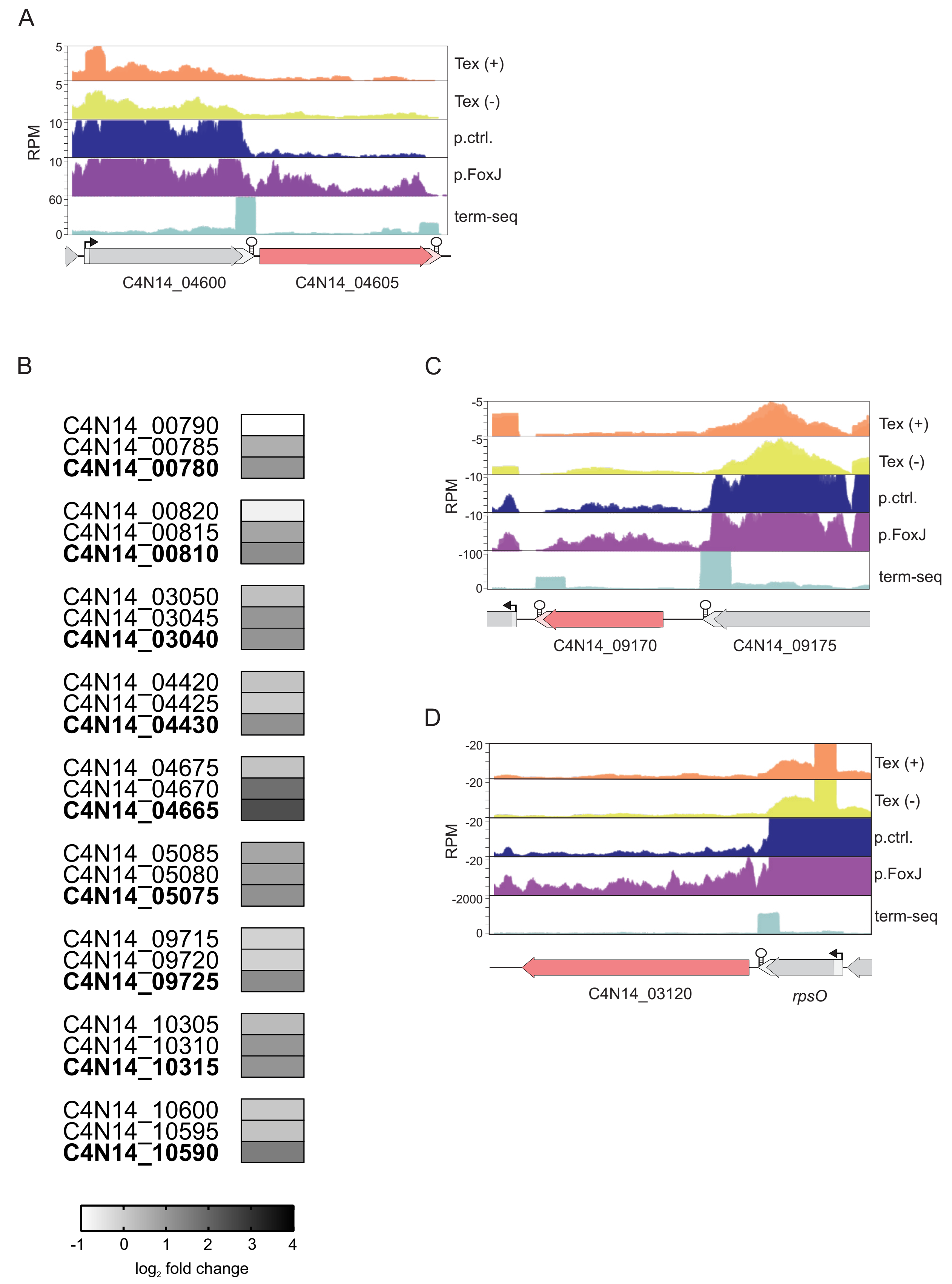

Fig. S6 Discordinate gene expression of operons with guanine-stretches in their 3' UTR.

(A) Normalized coverage (RPM) of the indicated RNA-seq data showing the read distribution for the positively regulated C4N14_04605 and surrounding genomic region. (B) Heatmap displaying the $\log_2$ fold changes of gene expression for operons that include at least one internal gene with a $\log_2$ fold change ≥ 1 when comparing the RNA-seq data of the FoxJ overexpression with that of the empty vector control (Fig. 5B). (C, D) Normalized coverage (RPM) of the indicated RNA-seq data showing the read distribution for the positively regulated C4N14_09170 (C) and C4N14_03120 (D) as well as their surrounding genomic region. Annotated TSS and TTS are indicated by an arrow and hairpin symbol, respectively.

Supplementary Fig. S7

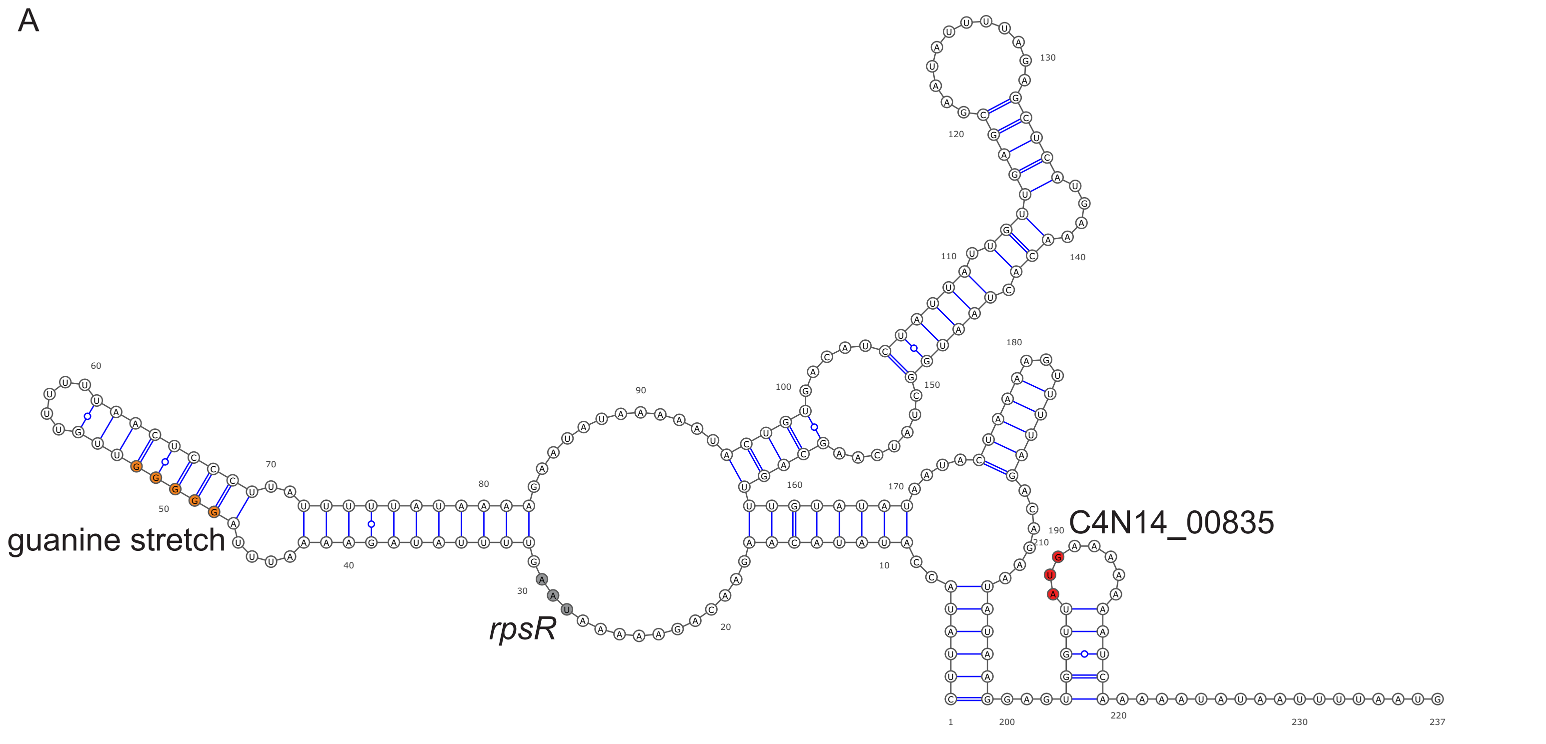

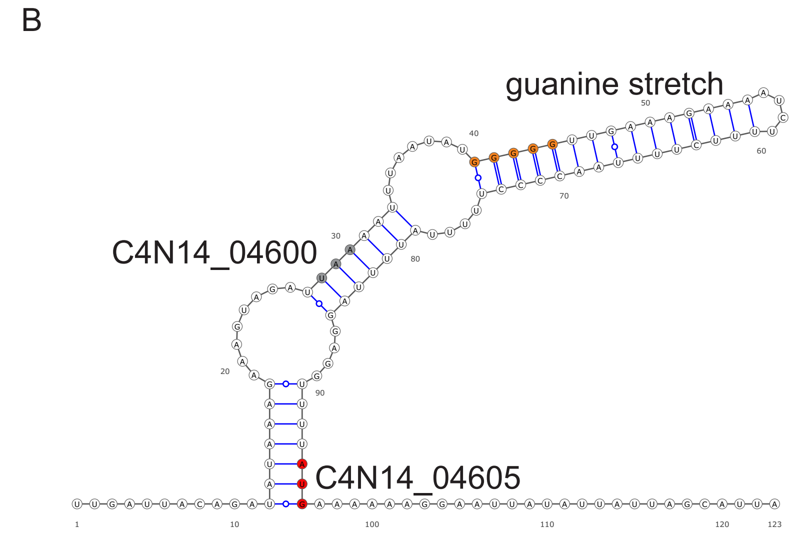

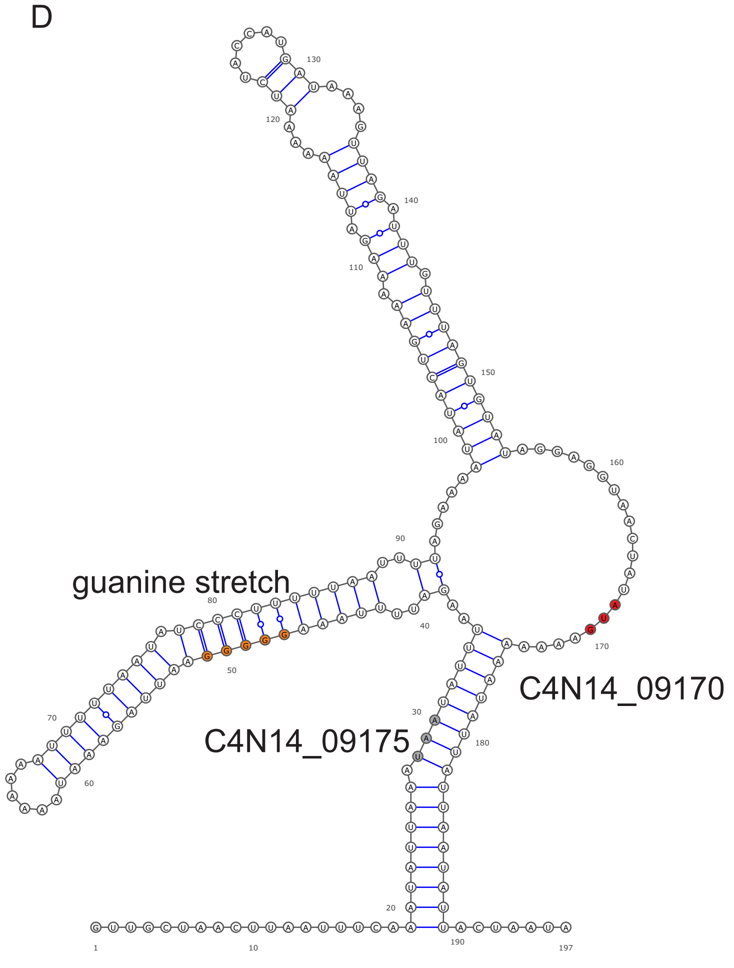

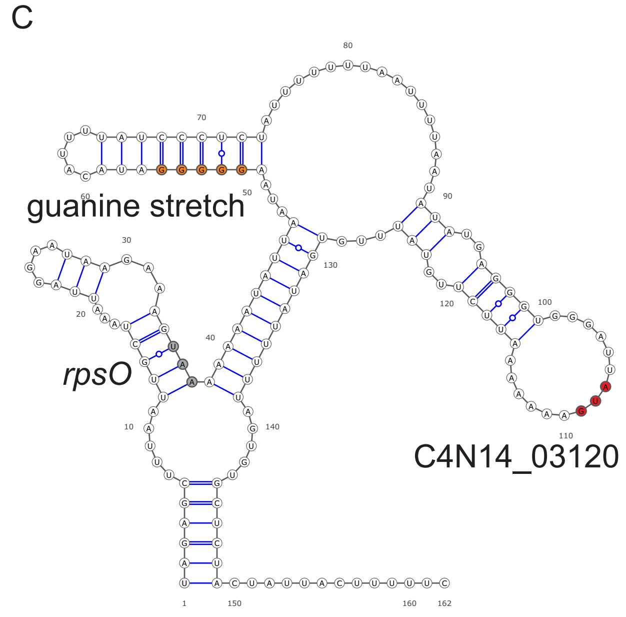

Fig. S7 Overview of predicted secondary structures of different IGRs with guanine stretches. Predicted secondary structure for the IGR of *rpsR*-C4N14_00835 (A), C4N14_04600-C4N14_04605 (B), *rpsO*-C4N14_03120 (C) and C4N14_09175-C4N14_09170 (D). The stop codons are marked in grey; the start codons are marked in red. The intrinsic terminator hairpins are labelled and the guanine-stretch is highlighted in orange.

Supplementary Fig. S8

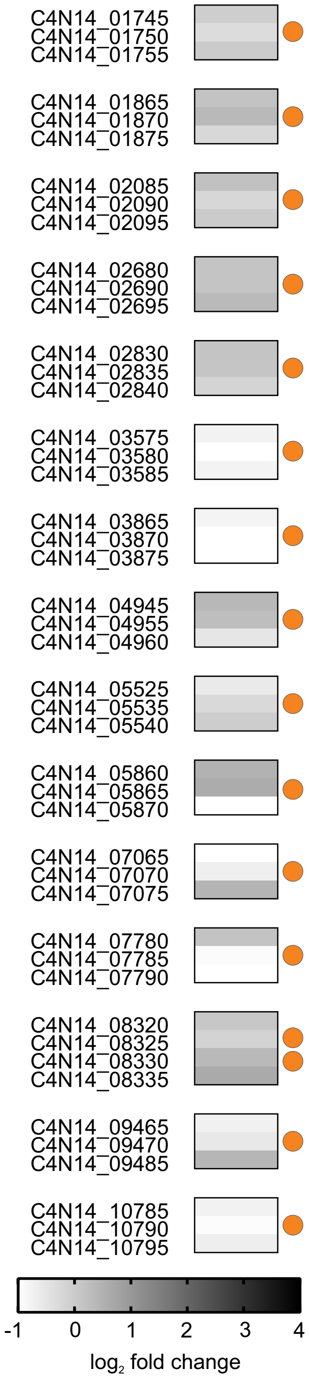

Fig. S8 Log$_2$ fold changes of additional genes carrying a guanine stretch in their 3' UTR.

Heatmaps displaying the log$_2$ fold changes for the gene expression upon FoxJ overexpression compared to the control. Genes harboring a guanine stretch in their 3' UTR, marked by an orange dot, are shown together with their respective up- and downstream gene.

Supplementary Fig. S9

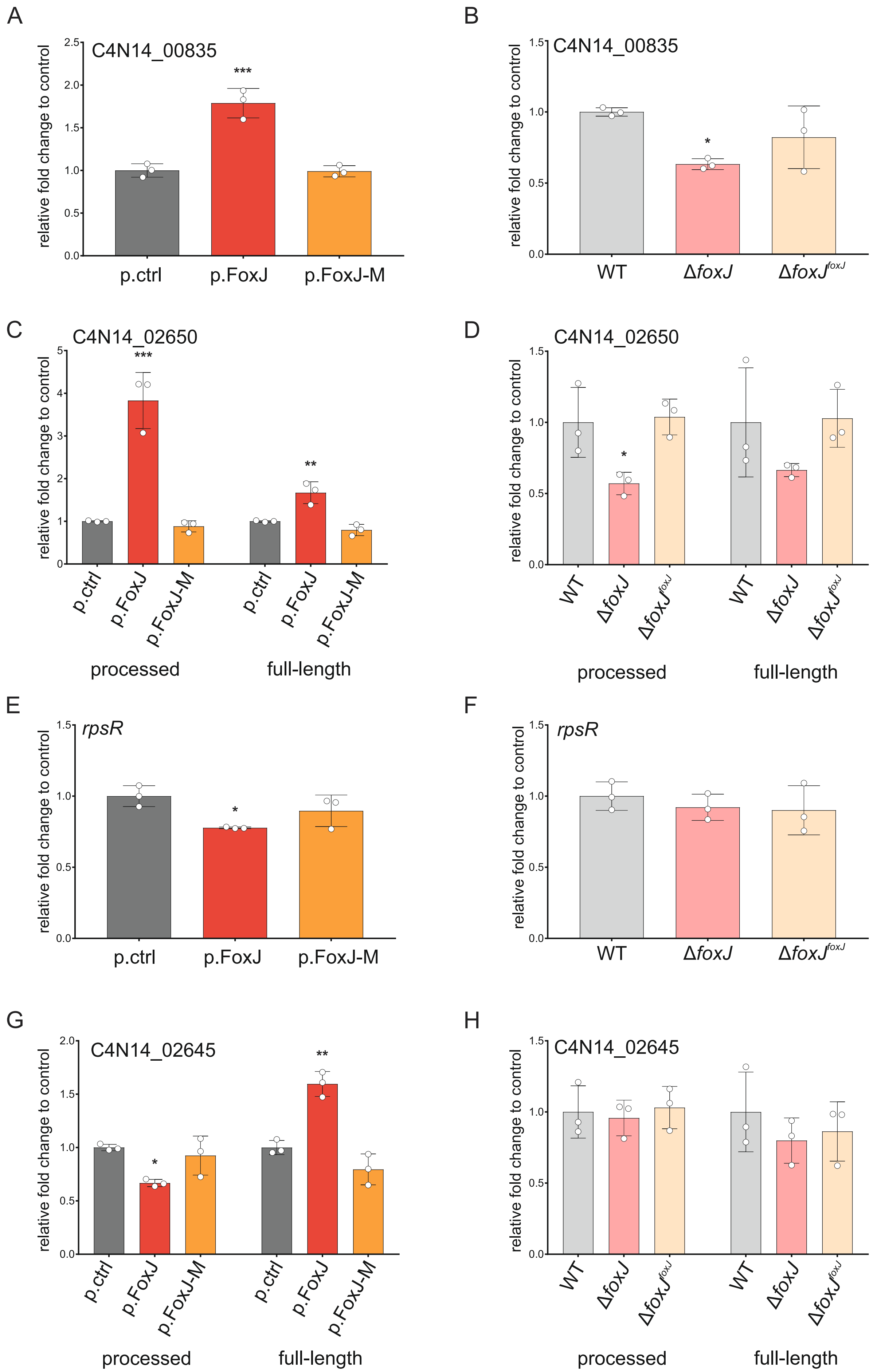

Fig. S9 Quantification of northern blots from Fig. 7.

Quantification of the northern blot signals from Fig. 7. (A) and (B) show the results when probing for the C4N14_00835 mRNA. (C) and (D) show the results when probing for the C4N14_02650 mRNA, measuring the intensity for either the processed band or the full-length one. (E) and (F) show the results when probing for the *rpsO* mRNA. (G) and (H) show the results when probing for the C4N14_02645 mRNA, measuring the intensity for either the processed band or the full-length one. The average of three biological replicates relative to that of the control (p.ctrl or WT) is displayed together with the standard deviation. Statistical testing was performed using a one-way ANOVA compared to the control group (p.ctrl or WT) (*: $P \leq 0.05$; **: $P \leq 0.01$; ***: $P \leq 0.001$; ****: $P \leq 0.0001$).
